# Evaluating the Antiproliferative Effects of Tri(2-Furyl)- and Triphenylphosphine-Gold(I) Pyridyl- and Pyrimidine-Thiolate Complexes

**DOI:** 10.3390/biom16010154

**Published:** 2026-01-15

**Authors:** Kyle Logan Wilhelm, Shyam Pokhrel, Drew Stolpman, Charli Worth, Sonal Mehta, Raul A. Villacob, Bernd Zechmann, Ahmad A. L. Ahmad, Joseph Taube, Mitchell R. M. Bruce, Alice E. Bruce, Touradj Solouki

**Affiliations:** 1 Department of Chemistry and Biochemistry, Baylor University, Waco, TX 76706, USA; kwilhelm@eastland.esc14.net (K.L.W.); drew2stolpman@gmail.com (D.S.); sonalm1298@gmail.com (S.M.); raul.villacob@bcm.edu (R.A.V.); 2 Department of Chemistry, University of Maine, Orono, ME 04469, USA; shyam.pokhrel@brescia.edu (S.P.); a.ahmad@jadara.edu.jo (A.A.L.A.); abruce@maine.edu (A.E.B.); 3 Department of Biology, Baylor University, Waco, TX 76798, USA; charli_worth1@baylor.edu (C.W.); joseph_taube@baylor.edu (J.T.); 4 Center for Microscopy and Imaging, Baylor University, Waco, TX 76798, USA; bernd_zechmann@baylor.edu

**Keywords:** auranofin, cancer, electrospray ionization (ESI), inductively coupled plasma mass spectrometry (ICP-MS), gold drugs

## Abstract

Two series of tri(2-furyl)- and triphenylphosphine-gold(I) complexes, with pyridyl- and pyrimidine-thiolate ligands containing electron-donating (-CH_3_) and electron-withdrawing (-CF_3_) substituents were synthesized and investigated for cell viability inhibitions. Prior results indicate that several of the gold(I) complexes in these series have high antifungal properties. The observed link between antifungal and anticancer activity provided motivation to investigate their antiproliferative effects, reported here. The synthesized compounds from both series were characterized by ^1^H, ^13^C, and ^31^P NMR spectroscopy, mass spectrometry (MS), infrared and UV-Vis spectroscopy, and solution stability studies. In addition, an X-ray crystallographic study was conducted on one of the gold(I) complexes. Analyte solubilities in McCoy’s 5A cell media were evaluated by ICP-MS. Initial screening studies were conducted on the two series to evaluate cell viability using the SK-BR-3 cell line. All ten gold(I) complexes exhibited sub-µM cytotoxicity and the most potent representatives, one from each series, were selected for further evaluation in four additional cell lines. Half-maximal effective concentrations (EC_50_) were determined for the MCF7 and MDA-MB-231 malignant mammary cell lines as well as the two control cell lines, HEK293T and MCF10A, to probe for specificity. Results indicate significant selectivity towards inhibition of cancer cells compared to non-transformed for tri(2-furyl)- and triphenylphosphine-gold(I) complexes with the 3,5-dimethylpyrimidine thiolate ligand when dissolved in cell media. Additional studies including 1% DMSO as a solubilizing agent revealed its significant impact on cellular responses.

## 1. Introduction

**Figure 1:**
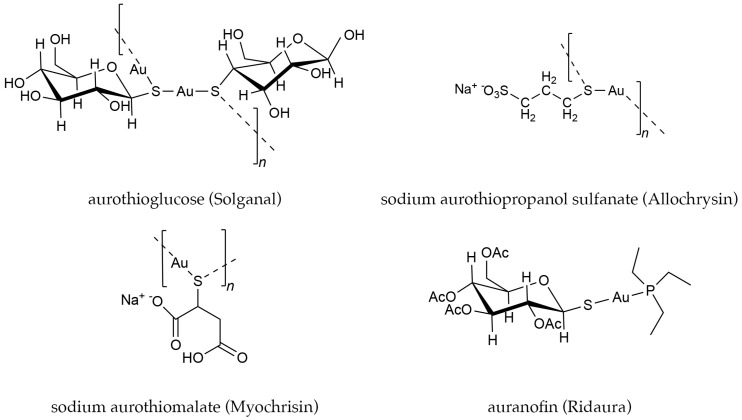
Examples of biologically active gold complexes.

Gold(I) thiolate complexes have been used to treat rheumatoid arthritis (RA), a chronic autoimmune disease that causes painful inflammation in the joints, since the early 1900s [1]. Early formulations of these drugs were water-soluble, injectable gold(I) salts, marketed under the common names Solganal, Allochrysin, and Myocrisin (Figure 1).

In 1985, the phosphine gold(I) thiolate complex, auranofin ((2,3,4,6- tetra-O-acetyl-1- thio-β-D-glucopyranosato-S)(triethylphosphine) gold) was approved by the Food and Drug Administration (FDA) as an oral medication for treating RA (Figure 1) [2,3]. The mechanism of the therapeutic effect of auranofin and other gold(I) drugs in the treatment of RA is still not completely understood. However, these drugs have been shown to have a wide range of medicinal effects, which has motivated numerous investigations into their possible treatment of other diseases, including cancer and parasitic diseases [4,5,6,7,8,9,10,11,12,13,14].

Recent efforts to repurpose auranofin for anticancer and antimicrobial properties have focused on mechanisms that induce apoptosis, including the inhibition of thioredoxin reductase [7,15,16,17,18,19]. Chemical proteomics analyses have confirmed the inhibitory effect of auranofin on TrxR and have also revealed effects on related pathways implicated in inflammation, glycogen metabolism, cellular differentiation, DNA replication, and selenoprotein synthesis [20,21]. A follow-up multifaceted proteome analysis to map redox-regulated cysteine residues revealed “massive changes in the redox state of the proteome” following a two-hour treatment with auranofin [22]. Interestingly, relative to the control, auranofin treatment resulted in a group of proteins that was oxidized and another group that was reduced. This intriguing result suggests that auranofin, and by extension, other phosphine gold(I) thiolate complexes, can participate in divergent but related pathways to increase reactive oxygen species (ROS), resulting in cell damage, and/or decrease ROS, which may ameliorate cell-damaging effects [4,23,24,25]. This complex response of the cell proteome to auranofin treatment makes mechanistic studies and the rational design of targeted gold-based drugs somewhat challenging, and suggests that a better understanding of these apparently divergent medicinal effects would be advantageous. These results also bring new insight into results from early clinical trials on treating RA with gold-based drugs, which showed that while 70–80% of patients experienced partial or complete remission of their arthritis, a significant number experienced side effects necessitating withdrawal from use [1].

**Scheme 1:**
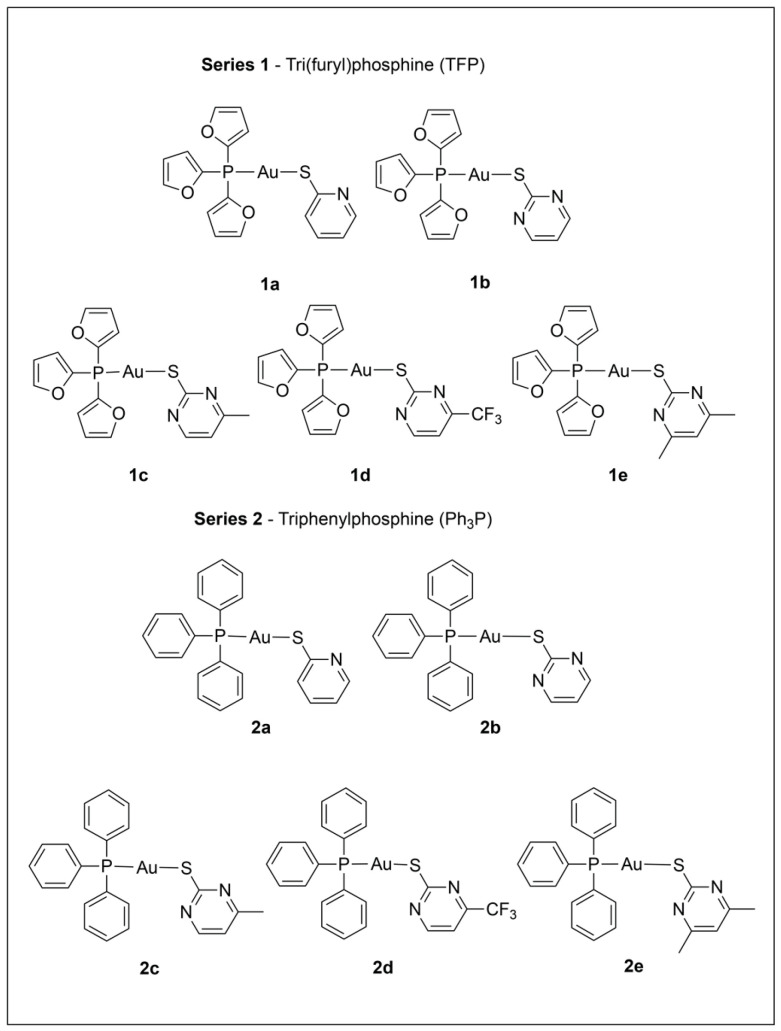
Numbering scheme and ligand abbreviations for the two series of gold(I) complexes studied. Abbreviations for thiolate ligands in the scheme caption: (**a**) Spy, (**b**) Spyrim, (**c**) SMepyrim, (**d**) SCF_3_pyrim, and (**e**) SMe_2_pyrim.

We recently investigated the antimicrobial properties of several of the tri(2-furyl)- and triphenylphosphine-gold(I) pyridyl- and pyrimidine-thiolate complexes depicted in Scheme 1, with **1b** and **2a** showing high antifungal activity (Minimum Inhibitory Concentration ≤ 1.25 µM) while not displaying cytotoxicity to mammalian cells at similar concentrations [26]. Several recent studies have noted a possible connection between antifungal and anticancer activity [27,28,29,30], and this provided additional motivation to investigate these gold complexes (Scheme 1) for their in vitro antiproliferative effects. Series 1 and Series 2 complexes in Scheme 1 contain tri(2-furyl) phosphine (TFP) and triphenylphosphine (PPh_3_), respectively. Relative to PPh_3_, TFP has a smaller cone angle and is a poorer π donor and a better π acceptor [31]. In addition, furyl rings vs. phenyl rings are expected to make the TFP ligand more polar. Each series includes the same set of thiolate ligands derived from pyridine and pyrimidine thiols, which are heterocyclic systems with a mobile hydrogen displaying tautomeric equilibria between the thiol and thione forms. These ligands, which contain sulfur and nitrogen donor atoms, are widely used in coordination chemistry for their versatile coordination behavior. Gold(I) is expected to preferentially bind to the sulfur [32], as is observed in Series 1 and 2. In addition, the nitrogen atoms in the ring can engage in hydrogen bonding [33,34], and have the potential to influence the hydrophilicity/lipophilicity balance of a metal complex, which is important for biological reactivity.

We recently investigated the antimicrobial properties of several of the tri(2-furyl)- and triphenylphosphine-gold(I) pyridyl- and pyrimidine-thiolate complexes depicted in Scheme 1, with **1b** and **2a** showing high antifungal activity (Minimum Inhibitory Concentration ≤ 1.25 µM) while not displaying cytotoxicity to mammalian cells at similar concentrations [26]. Several recent studies have noted a possible connection between antifungal and anticancer activity [27,28,29,30], and this provided additional motivation to investigate these gold complexes (Scheme 1) for their in vitro antiproliferative effects. Series 1 and Series 2 complexes in Scheme 1 contain tri(2-furyl) phosphine (TFP) and triphenylphosphine (PPh_3_), respectively. Relative to PPh_3_, TFP has a smaller cone angle and is a poorer π donor and a better π acceptor [31]. In addition, furyl rings vs. phenyl rings are expected to make the TFP ligand more polar. Each series includes the same set of thiolate ligands derived from pyridine and pyrimidine thiols, which are heterocyclic systems with a mobile hydrogen displaying tautomeric equilibria between the thiol and thione forms. These ligands, which contain sulfur and nitrogen donor atoms, are widely used in coordination chemistry for their versatile coordination behavior. Gold(I) is expected to preferentially bind to the sulfur [32], as is observed in Series 1 and 2. In addition, the nitrogen atoms in the ring can engage in hydrogen bonding [33,34], and have the potential to influence the hydrophilicity/lipophilicity balance of a metal complex, which is important for biological reactivity.

Herein, we report on the synthesis, characterization, and cell viability inhibitions of the phosphine gold(I) thiolate complexes shown in Scheme 1. The syntheses of **2a**, **2b**, **2d**, and **2e** were reported previously [35,36], and were included in this study to facilitate the comparison of two different phosphine ligands with an identical set of thiolate ligands. The structural characterization of these complexes is supported by NMR and IR spectroscopies, electrospray ionization mass spectrometry (ESI-MS), and X-ray crystallography data (for one member of Series 1). Moreover, we utilize inductively coupled plasma mass spectrometry (ICP-MS) to determine the accurate solubilities of these complexes in cell media. The study was conducted in two phases. In the first phase, we investigated the cytotoxic effects of Series 1 and 2, as well as auranofin and cisplatin controls, against the SK-BR-3 (ATCC HTB-30) breast cancer cell line. In the second phase, we identified a top candidate from each series and measured half-maximal effective concentrations (EC_50_) against the malignant mammary cell lines MCF7 (ATCC HTB-22) and MDA-MB-231 (ATCC HTB-26), as well as the mammary epithelial cell line MCF10A (ATCC CRL-10317) and the non-transformed fibroblast cell line HEK293T (ATCC CRL-11268). These four cell lines represent both hormone receptor-positive (MCF7) [37], and more aggressive triple-negative (MDA-MB-231) [38], breast cancer subtypes; moreover, MCF10A [39] and HEK293T [40] serve as non-malignant controls to evaluate the selectivity of the candidate gold compounds.

## 2. Materials and Methods

### 2.1. Materials

For syntheses of phosphine gold(I) thiolate complexes, reagent-grade chemicals, including acetonitrile, ether, ethyl acetate, methylene chloride, methanol, heptane, hexane, and pentane, were purchased from Thermo Fisher Scientific (Waltham, MA, USA). Deuterated chloroform (CDCl_3_) was purchased from Cambridge Isotope Laboratory Company (Tewksbury, MA, USA). Tri(2-furyl) phosphine, triphenylphosphine (99% pure), and triethylamine were purchased from Sigma Aldrich (St. Louis, MO, USA). 2-Mercaptopyridine (98%), 2-mercaptopyrimidine (98%), and 2-mercapto-4-methylpyrimidine hydrochloride (99%) were purchased from Alfa Aesar/Thermo Fisher (Tewksbury, MA, USA); 2-mercapto-4-(trifluoromethyl) pyrimidine (95%) and 4, 6-dimethyl-2-mercaptopyrimidine (98%) were purchased from Matrix Scientific (Elgin, SC, USA). Tetrahydrothiophene gold(I) chloride (Au(tht)Cl) was prepared according to the literature [41]. Cisplatin was purchased from Strem Chemicals (Newburyport, MA, USA), and auranofin was purchased from Enzo Life Sciences (Farmingdale, NY, USA). Trace metal nitric acid (HNO_3_, 70% *v*/*v*) and trace metal hydrochloric acid (HCl, 36% *v*/*v*) for ICP-MS sample preparation were purchased from Thermo Fisher Scientific (Waltham, MA, USA) and Sigma Aldrich (St. Louis, MO, USA), respectively. Nanopure water (18.2 MΩ) used for sample preparation was generated in-house using a Barnstead GenPure Pro Thermo Fisher Scientific (Waltham, MA, USA) Water Purification System. The ICP-MS external and internal calibration standards were purchased from Inorganic Ventures (Christiansburg, VA, USA). For cell viability studies, all five cell lines (SK-BR-3, MCF7, MDA-MB-231, HEK293T, and MCF10A) and FBS were purchased from ATCC (Manassas, VA, USA). Media components included the following: DMEM from Corning (Corning, NY, USA), DME-F12 from Cytiva (Marlborough, MA, USA), fetal bovine serum from ATCC (Manassas, VA, USA), horse serum from Cytiva (Marlborough, MA, USA), 1% penicillin/streptomycin from Gibco (Billings, MT, USA), 20 ng/mL EGF from EMD Millipore Corp (Burlington, MA, USA), 100 ng/mL chloera toxin from Sigma-Aldrich (St. Louis, MO, USA), 0.01 mg/mL insulin from Sigma-Aldrich (St. Louis, MO, USA), and 500 ng/mL hydrocortisone from ACROS (Geel, Belgium). CellTiter Blue and Trypan Blue dyes were purchased from Thermo Fisher Scientific (Waltham, MA, USA). Methanol (LC-MS grade) and acetic acid were also purchased from Thermo Fisher Scientific (Waltham, MA, USA). Sodium iodide calibrant was purchased from Waters (Milford, MA, USA). (Ph_3_P)AuCl and (TFP)AuCl [42] were prepared under N_2_ from equal molar mixtures of Au(tht)Cl and the respective phosphine in CH_2_Cl_2_ solution. The gold(I) products were isolated as powders by the addition of hexane; the formation of the desired products was confirmed by ^1^H, ^31^P{^1^H}, and ^13^C{^1^H} NMR spectroscopy.

### 2.2. Synthesis and Characterization of Phosphine Gold(I) Thiolate Complexes

All the phosphine gold(I) thiolate complexes shown in Scheme 1 (**1a**–**1e** and **2a**–**2e**) were prepared in a similar fashion. The simplified molecular input line entry (SMILE) notations for all ten gold compounds are listed in Table S1. Reactions were carried out under N_2_; isolation and recrystallization steps were conducted in the air. First, 0.54 mmol of thiol was dissolved in approximately 20 mL de-gassed CH_2_Cl_2_ at 0 °C, followed by the addition of NEt_3_ (90 µL, 0.65 mmol). The mixture was stirred until the solution became colorless and was transferred via cannula to a flask containing 0.54 mmol of (TFP)AuCl (for Series 1) or (Ph_3_P)AuCl (for Series 2) and stirred at room temperature (~23 °C) overnight. The reaction mixture was transferred to a separatory funnel, washed with 3 × 20 mL of water to remove triethylammonium chloride, and dried with anhydrous sodium sulfate. The solution was filtered and concentrated in vacuo. The addition of hexane precipitated the phosphine gold(I) thiolate complex. All complexes were recrystallized from CH_2_Cl_2_/heptane by slow evaporation of CH_2_Cl_2_. The tri(2-furyl) phosphine gold(I) thiolates (**1a**–**1e**) were obtained as pale-yellow crystals in 67–73% yield, and the triphenylphosphine gold(I) thiolates (**2a**–**2e**) were obtained as colorless crystals in 62–98% yield. Elemental analyses were determined by Galbraith Labs (Knoxville, TN, USA) (Table S2).

Melting points measured on a Capillary Melting Point Apparatus A388 from Thomas Hoover (Philadelphia, PA, USA), along with compound yields and spectral assignments, are available in Table S3.

NMR spectra were recorded on an Agilent/Varian Inova instrument (Santa Clara, CA, USA) and processed with MNova11 (Mestrelab Research, Santiago de Compostela, Spain); chemical shifts are reported in ppm. ^1^H NMR spectra (400 MHz) are referenced using the residual solvent peak of CDCl_3_ at 7.26 ppm. ^31^P{^1^H} NMR (162 MHz) spectra are externally referenced to 85% H_3_PO_4_ at (0 ppm). ^13^C{^1^H} NMR (101 MHz) spectra are referenced on the solvent, CDCl_3_ at 77.0 ppm (Figure S1). The same instrument was utilized for the collection of ^1^H and ^31^P NMR spectra to determine the stability of the complexes in DMSO-d_6_ over a 72 h time period (Figure S2).

The IR spectra were collected on solid samples using a Bruker ALPHA-P ATR spectrometer (Billerica, MA, USA) in the 4000–400 cm^−1^ wavenumber range (Figure S3).

### 2.3. X-Ray Crystallography

SC-XRD data were collected using a Bruker D8 QUEST ECO diffractometer (Madison, WI, USA) with a sealed tube source (Mo-Kα, λ = 0.71073 Å). Crystals of **1d** were obtained from methylene chloride/heptane. A crystal was mounted on a dual-thickness MiTeGen Micro Loop (MiTeGen, Ithaca, NY, USA). No decomposition or degradation of the crystal was observed during data collection, which was conducted at room temperature. The resulting structure was solved by direct methods using the APEX 3 Crystallography Software Suite version 6 (Bruker, Billerica, MA, USA). Further refinements were made with the XL refinement package in Olex2-1.5 [43,44]. Disorder in the CF_3_ group was modeled by assigning one-half occupancy to each fluorine part. All hydrogen atoms were calculated by the software and finally refined [43,44,45]. Cambridge Crystallographic Data Center deposition number for **1d**: 2184235 (Table S4).

### 2.4. ICP-MS

Analyses were carried out on Agilent ICP-MS 7900 (Santa Clara, CA, USA) to determine the solubility of the phosphine gold(I) thiolate complexes in McCoy’s 5A cell medium supplemented with 10% (*v*/*v*) fetal bovine serum (FBS). For solubility measurements, saturated solutions in McCoy’s 5A cell culture media (devoid of cells) for each complex were analyzed via ICP-MS. Concentrated hydrochloric and nitric acids were used to fully digest the saturated complex cell media solutions overnight at room temperature. The overnight digestion time was sufficient to fully dissolve all the gold in the solution, and no pressure- or microwave-assisted digestion was needed. Gold complexes were added to media individually in a microcentrifuge tube, sonicated for 60 min, and then shaken for 24 h to ensure each complex concentration surpassed its solubility limit; the presence of the undissolved complex as a visible pellet verified the saturation of the complex solution for ICP-MS analysis of the supernatant. Digestion of 500 µL aliquots of the saturated solution supernatant with 300 µL of HNO_3_ and 1000 µL of HCl was performed in 15 mL Eppendorf tubes overnight. The digested samples were properly diluted for analysis within the linear range of the ICP-MS. The Au standard (1002 ± 7 µg/mL) was diluted to produce an external calibration curve with ten concentrations from 1 to 10 ppb (ng/mL) (Figure S4), which was used to determine the concentration of Au in each solution and calculate the solubilities of each gold(I) complex in the cell culture media. All diluted digest samples, calibrants, continuing calibration verification samples (CCV), internal standards, and solvent blanks included 2.1% (*v*/*v*) HNO_3_ and 2.1% (*v*/*v*) HCl. All continuing calibration verification (CCV) data points were within 10% consistency throughout the entire ICP-MS data acquisition period, and hence no data points were rejected. The 50 ppb (ng/mL) Bi internal standard was used to correct for the drift in instrument response factor. Sample and calibration point intensities were solvent-blank-corrected after correcting for the internal standard. Each analyzed sample injection measured ^197^Au with seven 50-scan averages (0.5 s integration per scan). All internal standards and gold intensity (counts/s) relative standard deviations were within 2%, and no data points were rejected in the final ICP-MS analyses. Microsoft Excel (Version 2405) was used for ICP-MS data analysis and plotting.

### 2.5. LC-MS

Purities of **1e** and **2e** were determined with LC-MS and ESI-MS. To evaluate complex purities with LC-MS, each gold complex was dissolved in 18.2 MΩ water (to a final gold complex concentration of 0.1 µM), and 2 µL of each solution was injected onto a C18 trap and subsequent analytical columns (Waters ACQUITY UPLC M-Class HSS T3 C18, 100 µm × 150 mm, 100 Å pore size, 1.8 µm particle size). LC-MS mobile phase polarity was changed over time using a binary solvent system comprising Solvent A (18.2 MΩ H_2_O) and Solvent B (CH_3_CN). A 5 min isocratic trapping period (1% B) at a flow rate of 5 µL/min on the trap column was followed by a 45 min separation at an LC flow rate of 0.5 µL/min (Table S5). For the evaluation of stability, each complex was individually dissolved (99% 18.2 MΩ H_2_O: 1% CH_3_CN) to a final concentration of 0.5 µM and subjected to analysis after 72 h using the same LC-MS solvent composition and flow rates. Mass spectra were acquired using the Waters Synapt G-2 Traveling Wave Ion Mobility Spectrometry-Quadrupole-Time-of-Flight (Milford, MA, USA) system. All mass spectrometer settings were kept consistent with the ESI-MS section of this work. Data was collected in 1s scan times in the MS^E^ mode using an ion mass-dependent energy ramp of 10V–40V. Waters MassLynx software V4.2 (Milford, MA, USA) was utilized for data acquisition/analysis and control of instrument parameters. Microsoft Excel (Version 2405) and Inkscape (Version 0.92.3) were used for data plotting and visualization (Figure S5).

### 2.6. ESI-MS

Purities of phosphine gold(I) thiolate complexes were confirmed utilizing ESI-MS in positive-ion mode. Mass spectrometry data were monitored in real-time to ensure proper and consistent generation of ESI-generated ions. For all ESI-MS experiments reported here, a spray solution consisting of 99% 18.2 MΩ H_2_O and 1% CH_3_CN was used to replicate the initial LC-MS mobile phase composition. Each Au complex was dissolved in 99% H_2_O and 1% CH_3_CN and immediately placed in a pulled glass capillary for purity analysis. Data was collected over 8 s with an average scan time of 1 s. The ESI mass spectra (Figure S6) at 1.5 kV ionization source potential showed no major contaminants or reaction side products. Minor ion molecule reaction and rearrangement products, consistent with previously reported ESI mass spectra of gold(I) thiolate complexes [46,47], were observed.

All ESI-MS analyses were performed in the sensitivity (V) mode. Before each MS data acquisition, external calibration curves were generated using twelve sodium iodide ion clusters (Na_x_I_x−1_ where 14 ≥ x ≥ 2, excluding x = 3), which yielded mass measurement accuracies of better than 5 parts-per-million (ppm). The source parameters were kept constant, using the following settings: source temperature 40 °C, sampling cone 40 °C, source offset 80 V, backing pressure ~3 mbar, source pressure ~5 × 10^−3^ mbar, desolvation temperature 40 °C, purge nitrogen gas flow 100 mL/h, desolvation nitrogen gas flow of 100 L/h, and sample spray voltage 1.5 kV. All other instrument parameters were kept at the automatically optimized default settings.

### 2.7. UV-Vis

Stabilities of gold(I) complexes in cell media with 1% (*v*/*v*) DMSO were studied with a Thermo Varioskan LUX Multimode Microplate Reader (Waltham, MA, USA) equipped with SkanIt software (version 6.1). UV-Vis analyses required higher concentrations of gold(I) complexes, necessitating the use of DMSO to increase the complexes’ solubilities. Each gold(I) complex was dissolved in 1% DMSO and cell media to a final concentration of 5 µM for UV-Vis analysis. Each solution was added to a 96-well plate in triplicate. Each data set also included background spectra from twelve additional wells serving as blanks, including three empty wells, three wells with an equal volume of DMSO, three wells containing cell media, and three wells containing cell media with 1% DMSO. The average signal from the empty wells was subtracted from the average signal measured for all other wells. UV-Vis absorbance spectra were acquired from 250 to 500 nm immediately after sample preparation and after 72 h (Figure S7).

### 2.8. Cell Viability Measurements

All cell lines were obtained from commercial sources and have been used in similar studies [48,49,50]. Initial 3-point dose–response experiments with the SK-BR-3 cell line were conducted to screen the two series of complexes. These studies were conducted in the absence of DMSO. All cell counts were made with Trypan blue dye and a hemocytometer (Hausser Scientific, Horsham, PA, USA). Exponentially growing cells were seeded in 96-well microplates by adding 20,000 cells (200 cells per µL) to each well and subsequently allowing 24 h for the cell population to adhere to the well walls before the introduction of the treatment. For each cell viability analysis, 24 h after seeding, the medium in each well was replaced with the prepared treatment solutions of each complex. The 96-well plates were then incubated at 37 °C in a 5% CO_2_ atmosphere for an additional 72 h. Auranofin (0.5 µM) and cisplatin (5 µM) were used as positive controls.

After obtaining the initial screening cell viability measurements, **1e** and **2e** were selected for further study for more in-depth cell viability assessments on the MCF10A, HEK293T, MDA-MB-231, and MCF7 cell lines, with solutions made by direct dissolution of **1e** and **2e** in cell media across the target concentrations (viz., 0.01 µM, 0.03 µM, 0.06 µM, 0.1 µM, 0.33 µM, 0.66 µM, 0.75 µM, and 1 µM), along with a second analogous solution set incorporating a nominal volume (1% *v*/*v*) of dimethyl sulfoxide (DMSO) in cell media to study the effects of DMSO on cell viability experiments. These four solution sets (**1e** without DMSO, **1e** with DMSO, **2e** without DMSO, and **2e** with DMSO) were employed in cell viability analyses over a 72 h incubation period. The observation period of 72 h is a conventional practice in evaluating gold(I) complexes and is attributed to the protracted presence of gold in the body post-treatment [51,52,53,54]. Three types of control wells were designated as follows: the blank control, which contained only cell media without any gold complex or cells added, the control population, which contained cells without any drug or DMSO, and the final control, which contained the same number of cells and the same percentage of DMSO (1% *v*/*v*) without any gold complex added. These controls were included to account for the natural absorbance of the cell media, measure the maximum fluorescence anticipated for the initial number of cells unperturbed, and account for the anticipated cell morbidity caused by the presence of DMSO. Auranofin was included in the cell viability assays to validate the observed cellular responses; cell viability measurements for the auranofin control were consistent with previously reported values [48,50]. CellTiter Blue was utilized for cell viability measurements, following the manufacturer’s recommended volumes and incubation times. After adding the CellTiter Blue and incubating the plate for four hours (37 °C, 5% CO_2_), fluorescent measurements were made of each well with respective excitation and emission wavelengths of 560 nm and 590 nm. The relative intensity of the control populations in each well was accounted for, and measurement values were adjusted accordingly. This process was completed in triplicate for each treatment regime. Determination of EC_50_ was derived from eight-point dose–response curves spanning concentrations from 0.01 µM to 1 µM for each of the four solution sets. Each dose response was fit to a sigmoidal function using GraphPad Prism 10.3.0 (San Diego, CA, USA), with all the treatments producing R^2^ values of ≥0.98 and relative standard deviations ≤ 10% between sample replicates (Figure S8).

## 3. Results and Discussion

### 3.1. Characterization and Assessment of Purity for Series 1 and 2

In this study, we report on a small, targeted library consisting of the two series of gold(I) complexes shown in Scheme 1 that incorporate subtle variations in the steric and electronic properties of the phosphine and thiolate ligands. The complexes in Series 1 and Series 2 contain tri(2-furyl) phosphine (TFP) and triphenylphosphine (PPh_3_), respectively, and the same set of thiolate ligands: (**a**) Spy, (**b**) Spyrim, (**c**) SMepyrim, (**d**) SCF_3_pyrim, and (**e**) SMe_2_pyrim. The thiolate ligands, which are derived from pyridine and pyrimidine thiols, vary in terms of the number and placement of substituent groups on the ring (e.g., electron-donating -CH_3_ and electron-withdrawing -CF_3_ groups). The study design included assessing the solubility of all ten gold complexes in cell media and an initial screening for relative cytotoxicity against a single breast cancer cell line, SK-BR-3. This information was then used to identify one candidate in each series for further evaluation against two additional malignant mammary cell lines (MCF7 and MDA-B-231) as well as two control cell lines (a normal breast cell model, MCF10A, and human embryo kidney cells, HEK293T) to probe for specificity.

The gold(I) complexes were prepared by reaction of equimolar amounts of the deprotonated thiol, and (TFP)AuCl (Series 1) or (Ph_3_P)AuCl (Series 2). Prior to conducting cell viability experiments, all ten gold complexes were characterized by ^1^H, ^13^C{^1^H}, ^31^P{^1^H} NMR spectroscopy (Figure S1), FTIR spectroscopy (Figure S3), ESI-MS (Figure S6), and thermal conductivity detector-based elemental analysis (Table S2). The S-pyridine and S-pyrimidine ligands typically display a variety of coordination modes with transition metals, but with gold(I), they most frequently behave as anionic ligands coordinated in a monodentate fashion through the sulfur donor atom. The FTIR spectra for Series 1 and Series 2 are consistent with this mode of coordination (Figure S3). The absence of ν(N-H) near 3150–3050 cm^−1^ and the absence of ν(S-H) (which generally appears as a broad, weak band between 2600 and 2400 cm^−1^) are consistent with deprotonation of the S-pyridine and S-pyrimidine molecules and formation of thiolate ligands coordinated to gold(I). The ^1^H and ^13^C{^1^H} NMR spectra (Figure S1) are also consistent with gold(I) being coordinated to one phosphine and one thiolate ligand. All complexes display a single sharp resonance in their ^31^P{^1^H} NMR spectra (Figure S1), with the TFP (in **1a**–**1e**) and Ph_3_P (in **2a**–**2e**) resonances appearing at −23.4 to −24.2 ppm and 38.1 to 38.3 ppm, respectively. The ^31^P{^1^H} NMR spectral resonances for Series 1 and 2 complexes are shifted several ppm downfield relative to (TFP)AuCl (−29 ppm) and (Ph_3_P)AuCl (31 ppm), respectively, consistent with the substitution of a chloride ligand by thiolate [35,55,56]. The purity of the complexes was established by elemental analyses (Table S2) and ESI-MS (Figure S6). Experimental values for the elemental analyses of C, H, and N were within 0.4–0.6% of their calculated values. The ESI mass spectra at 1.5 kV ionization source potential showed no major contaminants or reaction side products. Moreover, experimentally observed isotopic patterns and measured masses agreed with their respective theoretical values (Figure S6). Specifically, calculated mass measurement errors for all gold complexes were less than 5 ppm, in agreement with the elemental analysis results (Table S2).

### 3.2. X-Ray Structure

**Figure 2:**
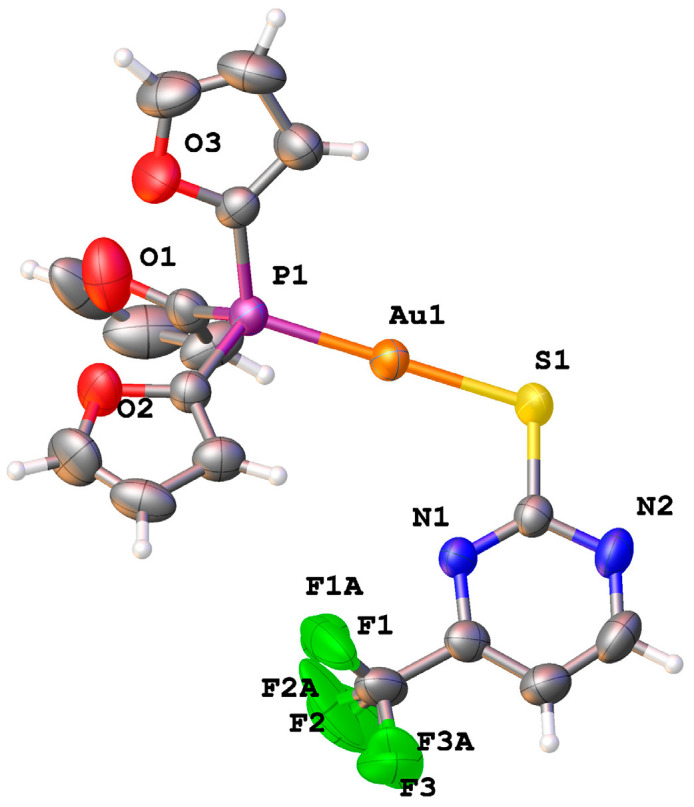
ORTEP diagram (50% thermal ellipsoids) for **1d** (TFP)Au(SCF_3_pyrim). Color scheme: C (gray); N (blue); O (red); P (purple); S (yellow); F (green); Au (orange).

Complex **1d** was characterized by single-crystal X-ray diffraction. Figure 2 shows the molecular structure for **1d** displayed as an Oak Ridge Thermal Ellipsoid Plot (ORTEP) diagram, and crystal data are provided in Table S4. The Au-P (2.2451(14) Å) and Au-S (2.2927(14) Å) bond distances and the P-Au-S angle (178.35(6)°) are typical for phosphine gold(I) thiolate complexes. The CF_3_ group exhibits positional disorder in the F atoms. The intramolecular Au-N distance of 3.226(4) Å is close to the sum of the Au-N van der Waals radii (3.21 Å); however, Au-N interactions do not appear to contribute significantly to the structural properties, given the linear geometry at gold.

### 3.3. Solubility and Stability of Series 1 and 2 in Cell Media

The solubility of each complex in McCoy’s 5A cell medium supplemented with 10% (*v*/*v*) FBS was determined using ICP-MS. The known gold–complex stoichiometry of 1:1 and quantitative determination of gold concentrations in media saturated with each complex were utilized to determine the solubility of each complex in separate experiments (Table 1). The ICP-MS calibration curve (Figure S4) indicated a high degree of linearity (e.g., R^2^ = 0.9995). Based on the replicate ICP-MS measurements and including error propagations, relative uncertainties for solubility values at the 95% confidence level (CL = 95%, n = 48) were less than 2% (Table 1). Solubilities for the examined gold(I) complexes ranged from ~1.07 µM (for **2b**) to ~36.8 µM (for **1b**); auranofin’s solubility in the cell culture medium at the 95% confidence level (95%) was 43.6 ± 0.5 µM. On average, gold complexes with the TFP ligand exhibited slightly greater solubility (average 14.8 µM) than the PPh_3_ complexes (average 4.6 µM), consistent with the greater degree of hydrophilicity for the furyl vs. phenyl rings.

Stability of the gold complexes in cell media over a 72 h time frame was assessed by UV-Vis spectroscopy. Since UV-Vis analyses require higher concentrations, 1% (*v*/*v*) DMSO was added to achieve 5 μM concentrations of the gold complexes. All UV-Vis absorbance spectra were acquired immediately after sample preparation and compared with corresponding data collected after 72 h. No significant changes were observed in the UV-Vis spectra during the 72 h period (Figure S7).

### 3.4. Phase 1: Cytotoxicity of Series 1 and 2 Against the SK-BR-3 Breast Cancer Cell Line

The initial cytotoxicity screening of Series 1 and 2 was made against the breast cancer cell line, SK-BR-3, at three concentrations (viz., 0.125, 0.25, and 0.5 µM). The cell viability measurements were acquired using a fluorescent assay (CellTiter Blue), and all cell viability results were normalized based on the fluorescence observed from the control samples. The results shown in Table 2 demonstrate that all 10 complexes show antiproliferative activity at 0.5 µM concentrations. The observed respective average cell viabilities against SK-BR-3 for Series 1 and 2 were 59 ± 5% and 49 ± 8%. Auranofin (0.5 µM) and cisplatin (5 µM) were included as positive controls against SK-BR-3, and these complexes exhibited cell viability reductions of 47.1 ± 1.3% and 49.9 ± 3.8%, respectively, consistent with the literature values [15,16]. Further, complexes **2b**, **2d**, and **2e** showed cell viability reductions larger than 50% (viz., 100% − 48.5% ≅ 52.5%, 100% − 49.9% ≅ 50.1%, and 100% − 48.7% ≅ 52.3%, respectively) at the 0.5 µM concentration level.

**Figure 3:**
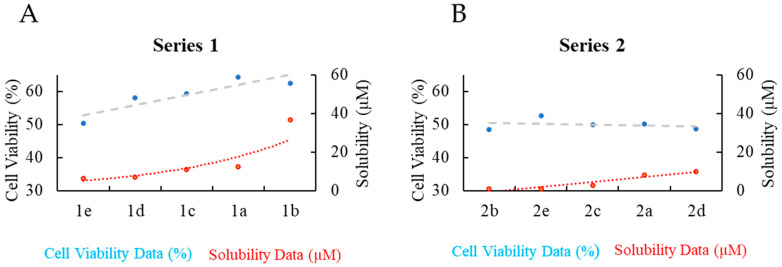
Dual-axis charts showing cell viability results for SK-BRK-3 (blue circles) and solubility in cell media (red circles) for (**A**) Series 1 and (**B**) Series 2; sorted by solubility. Dotted lines are trendlines.

To examine the differences in activity between Series 1 and 2 in more detail, the cell viability and solubility data were plotted in the dual-axis charts shown in Figure 3. There is a wider range of cell viability for Series 1 (50.4 ± 1.9 to 64.4 ± 1.1%) than Series 2 (48.5 ± 1.7 to 51.9 ± 7.0%). For Series 1, there is a trend between solubility and cell viability, with the less soluble complexes showing higher cytotoxicity (Figure 3A). Series 2 is, on average, more cytotoxic and less soluble than Series 1, but there is little variation in either property within the series (Figure 3B). Solubility in aqueous solution is expected to be inversely correlated with lipophilicity [57,58], and the balance between lipophilicity and hydrophilicity plays an important role in the transport of molecules through biological membranes [59,60]. McKeage et al. investigated the correlation between lipophilicity and cytotoxicity for a series of cationic gold(I) complexes, and they found a parabolic dependence between lipophilicity (log Kw) and logarithmic IC_50_ values, where the activity reached a maximum at an intermediate value of log Kw. Moreover, there was a linear relationship between cellular uptake and logarithmic IC_50_ values. Additional studies have shown a parabolic relationship between antitumor activity and drug lipophilicity, with the highest activity occurring at an intermediate value of lipophilicity [59,61,62]. The data shown in Figure 3 suggest that differences in cytotoxicity between Series 1 and 2, and within Series 1, may be related to slight differences in cellular uptake due to the subtle influence of ligand substituents on the hydrophilic–lipophilic balance of the complexes. Based on these SK-BR-3 breast cancer cell viability results, complexes **1e** and **2e** (the closest analog of **1e** in series 2) were selected for additional cell viability studies in phase 2.

### 3.5. Phase 2: Cytotoxicity and Selectivity of **1e** and **2e** for MDA-MB-231 and MCF7 Breast Cancer Cell Lines

Prior to conducting additional cell studies, the purity and stability of **1e** and **2e** were further evaluated with LC-MS and NMR spectroscopy. Based on the observed LC peaks (Figure S5) and direct infusion MS data (Figure S6), both complexes were >95% pure. LC-MS data confirmed the absence of impurities and the presence of expected ion-molecule reaction products within the parent molecules’ LC elution times. Hence, LC-MS results were consistent with the IR and NMR spectroscopy data, and other analytical characterizations (vide infra) in confirming the purity of **1e** and **2e**. Using an optimized solvent gradient (Table S5), ultra-performance LC (UPLC) ESI-MS analyses of **1e** and **2e** dissolved in 99% 18.2 MΩ H_2_O: 1% CH_3_CN to a final concentration of 0.5 µM (Figure S5) confirmed stability over 72 h, ≥95% purity, and correct chemical identities of the examined gold compounds. Finally, in addition to the UV-Vis analyses described above for the evaluation of stability of Series 1 and 2 in cell media with 1% (*v*/*v*) DMSO added, the stability of **1e** and **2e** in neat DMSO-d_6_ was evaluated by ^1^H and ^31^P{^1^H} NMR spectroscopy, which confirmed the stability of the complexes over a 72 h testing period (Figure S2), i.e., no spectral changes were noted in any of the NMR spectra as a function of time.

In phase 2, cell viability assessments for **1e** and **2e** were conducted on four cell lines, including two mammary malignant cell lines (MCF7, MDA-MB-231), one mammary epithelial cell line (MCF10A), and one non-transformed fibroblast cell line (HEK293T). Two different solution sets were prepared for each cell viability assessment: one set with the direct dissolution of complexes in cell media across the target concentrations (0.01 μM to 1 μM) and another analogous solution set that included 1% *v*/*v* DMSO to enhance the solubility of **1e** and **2e**. These two sets of experimental conditions were used to evaluate the influence of DMSO on the cellular responses. Although DMSO is commonly employed to prepare treatment solutions of organic and inorganic compounds with low aqueous solubility, there is evidence in the recent literature that DMSO can influence the cell viability results [63,64,65,66,67,68]. Other studies have shown that impurities in DMSO may complicate reactivity studies involving metal ions [69]. Although the concentration of DMSO in many cell viability studies is typically no more than 1%, several studies suggest that concentrations greater than 0.3% may have detrimental effects. Other studies conclude that blank control experiments containing the same level of DMSO as the test solutions are necessary and sufficient to establish the effect of DMSO [49].

The results of EC_50_ studies against four cell lines, with direct dissolution of **1e** and **2e** in cell media and in the absence of DMSO, are shown in Table 3. The eight-point dose–response curves, spanning a concentration range of 0.01 µM to 1 µM, which were used for the determination of EC_50,_ are shown in Figure S8A,C. EC_50_ values, derived from sigmoidal fits of the dose–response curves, range from 0.05 ± 0.01 µM for **2e** against MDA-MB-231 to 0.31 ± 0.03 µM for **1e** against MCF7. Further, dose–response data indicate that, regardless of the concentration level, treatments of non-transformed epithelial (MCF10A) and fibroblast cells (HEK293T) with **1e** or **2e** resulted in mostly unperturbed cell populations (cell viabilities greater than 80%). Thus, **1e** and **2e** exhibited a significant selectivity towards inhibition of the cancer cell lines as compared to non-transformed cells. Auranofin has been tested against the MCF7 and MDA-MB-231 cell lines in numerous studies, without DMSO or with low levels of DMSO, with IC_50_ values in the range of 0.6–1.5 µM and 0.47–1.0 µM for MCF7 and MDA-MB-231, respectively [50,70,71,72]. It is noteworthy that **1e** and **2e** appear to be more active than auranofin against these two breast cancer cell lines.

### 3.6. Further Discussion on the Influence of 1% DMSO on the Cytotoxicity and Selectivity of **1e** and **2e** for MDA-MB-231 and MCF7 Breast Cancer Cell Lines

The second set of cytotoxicity studies on the same four cell lines was carried out for **1e** and **2e** dissolved in cell media containing 1% (*v*/*v*) DMSO. Control experiments on our cell populations treated with cell media containing 1% DMSO (i.e., without any gold complexes) establish that there is no significant reduction in cell viability for any of the four cell lines (≤10% cell viability reduction). Further, the gold(I) complexes in Series 1 and 2 are stable in cell media with 1% DMSO as well as in neat DMSO solutions, ruling out the possibility of **1e** and **2e** reacting with DMSO to form decomposition products.

The results of EC_50_ studies against the four cell lines in the presence of 1% DMSO and the gold complexes **1e** and **2e** (see Table 4) indicate significantly altered results. A comparison of the data in Table 3 and Table 4 suggests that **1e** and **2e** are more cytotoxic to MCF7 in the presence of 1% DMSO than in the absence of DMSO. The opposite is true for the MDA-MB-231 cell line, where **1e** and **2e** are more cytotoxic in the absence of DMSO. Beyzavi et al. reported cell viability studies (with 1% DMSO) on a related series of gold(I) thiolate complexes that included the diphenyl-2-pyridylphosphine ligand and similar thiolates to those in Series 1 and 2. In their studies, the gold complexes with the Spy and Spyrim ligands had an average IC_50_ concentration of 6.7 µM for the MCF-7 cell line following a 72 h treatment period [73]. In contrast, our results on a similar set of gold(I) complexes, **1e** and **2e**, demonstrate EC_50_ concentrations against the MCF7 cell line that are an order of magnitude lower after a 72 h treatment.

Significantly, the results shown in Table 4 indicate considerable toxicity for **1e** and **2e** toward the noncancerous cell lines, MCF10A and HEK293T, in the presence of 1% DMSO. What might be responsible for this effect? It is interesting to note that DMSO can influence the fluidity and permeability of biological membranes [74,75,76]. In addition, studies have demonstrated that certain ovarian cancer cells (IGROV1), which are known to have greater membrane fluidity, are more sensitive to auranofin treatment [77]. Taken together, our data suggest that the presence of 1% DMSO in the cell media may alter the cell membranes in some way that increases the toxicity of **1e** and **2e** toward the non-transformed cell lines. These results provide a cautionary note that the combination of DMSO and active compounds may change their mechanisms of cytotoxicity.

## 4. Conclusions

Based on prior results that indicated that **1b** and **2a** show very high antifungal activity while not displaying cytotoxicity to mammalian cells at similar concentrations, ten phosphine gold(I) complexes with two different phosphine ligands (TFP and PPh_3_) and five different pyridine and pyrimidine thiolate ligands were synthesized and evaluated for antiproliferative activities. The purity and stability of the synthesized gold(I) complexes were determined with NMR and IR spectroscopies, elemental analysis, ESI-MS, and LC-MS. Complex **1d** was also characterized by single-crystal X-ray diffraction. Their solubility in aqueous cell media was also determined.

The complexes with the SMe_2_pyrim ligand, **1e** and **2e**, exhibited high cytotoxicity against the SK-BR-3 cell line, and these two complexes were selected for additional evaluation against four cell lines. The results of EC_50_ studies in cell media with no added DMSO indicate significant selectivity towards inhibition of the two mammary malignant cell lines, MCF7 and MDA-MB-231, as compared to the two non-transformed cells, MCF10A and HEK293T. Further, the data indicate that, regardless of the concentration level, neither the **1e** nor **2e** complexes reduced cell viability below 80% in the treatments of the noncancerous mammary epithelial cell line (MCF10A) or the non-transformed fibroblast cell line (HEK293T). In parallel studies conducted with 1% DMSO, both **1e** and **2e** inhibited the proliferation of the cancerous and non-transformed cells with minimal differences.

The data presented in this study strongly suggest the need for incorporating dual cell viability experiments into future investigations involving gold complexes. Specifically, these experiments should encompass both DMSO-assisted and DMSO-free conditions during the solvation of complexes. Such a comprehensive approach will offer valuable insights into the underlying reasons for the observed impact of DMSO on cell viability results. Furthermore, this methodology will enable the identification and exclusion of complexes that exhibit inherent insolubility at target concentrations, thus refining the selection of candidates for future studies. This dual experimental design, comparing the effects of the complexes with and without DMSO, will contribute to the establishment of robust protocols for assessing the cytotoxic potential of gold complexes and enhance the reliability and reproducibility of results in this field. Thus, it is important for future studies to consider the solubility limits of potential drug candidates and carefully assess the influence of DMSO on the measured cell viability outcomes.

## Figures and Tables

**Table 1:** Solubility limits for gold complexes and auranofin in McCoy’s 5A cell medium at the 95% confidence level, determined by quantification of gold in replicate ICP MS measurements.

Complex	Solubility Limit (µM)
**1a**; (TFP)Au(Spy)	12.5 ± 0.0_9_
**1b**; (TFP)Au(Spyrim)	36.8_0_ ± 0.5_0_
**1c**; (TFP)Au(SMepyrim)	11.0_4_ ± 0.0_9_
**1d**; (TFP)Au(SCF_3_pyrim)	7.0_7_ ± 0.0_6_
**1e**; (TFP)Au(SMe_2_pyrim)	6.5_9_ ± 0.0_6_
**2a**; (Ph_3_P)Au(Spy)	8.2_0_ ± 0.1_0_
**2b**; (Ph_3_P)Au(Spyrim)	1.0_7_ ± 0.0_2_
**2c**; (Ph_3_P)Au(SMepyrim)	2.9_6_ ± 0.0_4_
**2d**; (Ph_3_P)Au(SCF_3_pyrim)	10.0_0_ ± 0.0_1_
**2e**; (Ph_3_P)Au(SMe_2_pyrim)	1.1_2_ ± 0.0_2_
Auranofin	43.6 ± 0.5_0_

**Table 2:** Individual cell viability results for SK-BR-3 after incubation of **1a**–**1e** and **2a**–**2e** in McCoy’s 5A media for 72 h (0.5 µM). For comparison, auranofin (0.5 µM) and cisplatin (5 μM) results are also included in the last two rows. All cell viability values are reported at the 95% confidence level (for *n* = 15).

Complex	Cell Viability (%)
**1a**; (TFP)Au(Spy)	64._4_ ± 1._1_
**1b**; (TFP)Au(Spyrim)	62._5_ ± 2._7_
**1c**; (TFP)Au(SMepyrim)	59._3_ ± 2._5_
**1d**; (TFP)Au(SCF_3_pyrim)	58._1_ ± 2._5_
**1e**; (TFP)Au(SMe_2_pyrim)	50._4_ ± 1._9_
**2a**; (Ph_3_P)Au(Spy)	50._1_ ± 2._0_
**2b**; (Ph_3_P)Au(Spyrim)	48._5_ ± 1._7_
**2c**; (Ph_3_P)Au(SMepyrim)	51._9_ ± 7._0_
**2d**; (Ph_3_P)Au(SCF_3_pyrim)	49._9_ ± 2._3_
**2e**; (Ph_3_P)Au(SMe_2_pyrim)	48._7_ ± 1._7_
Auranofin	52._9_ ± 1._3_
Cisplatin	50._1_ ± 3._8_

**Table 3:** EC_50_ values for **1e** and **2e** dissolved in cell media; concentrations expressed in µM for each solution after 72 h.

Cell Line	1e (EC_50_ (µM))	2e (EC_50_ (µM))
MDA-MB-231(breast cancer)	0.07 ± 0.01	0.05 ± 0.01
MCF7 (breast cancer)	0.31 ± 0.03	0.11 ± 0.02
MCF10A (non-transformed)	Cell Viability > 80%	Cell Viability > 80%
	at doses ≥ 0.3–1.0 µM	at doses ≥ 0.3–1.0 µM
HEK293T (non-transformed)	Cell Viability > 80%	Cell Viability > 80%
	at doses ≥ 0.3–1.0 µM	at doses ≥ 0.3–1.0 µM

**Table 4:** EC_50_ values for **1e** and **2e** dissolved in cell media and with 1% DMSO added; concentrations expressed in µM for each solution after 72 h.

Cell Line	1e (EC_50_ (µM) in 1% DMSO)	2e (EC_50_ (µM) in 1% DMSO)
MDA-MB-231 (breast cancer)	0.16 ± 0.02	0.19 ± 0.02
MCF7 (breast cancer)	0.07 ± 0.01	0.08 ± 0.01
MCF10A (non-transformed)	0.09 ± 0.01	0.15 ± 0.01
HEK293T (non-transformed)	0.20 ± 0.02	0.32 ± 0.03

## Data Availability

The original contributions presented in this study are included in the article/Supplementary Materials. Further inquiries can be directed to the corresponding authors.

## Supplementary Materials

The following supporting information can be downloaded at: https://www.mdpi.com/article/10.3390/biom16010154/s1, Figure S1: ^1^H, ^13^C, and ^31^P NMR spectra for **1a**–**1e**; **2a**–**2e** in **CDCl3**; Figure S2: ^1^H, and ^31^P NMR 72-h spectra for **1e** and **2e**; Figure S3: Attenuated total reflectance (ATR)-FTIR spectra for **1a**–**1e**; **2a**–**2e**; Figure S4: Calibration curve for ICP-MS analyses of gold concentrations; Figure S5: UPLC-ESI-MS data for **1e** and **2e**; Figure S6: Positive-ion mode ESI-MS mass spectra for **1a**–**1e**; **2a**–**2e**; Figure S7: UV-Vis spectra for **1a**–**1e** and **2a**–**2e**; Figure S8: Eight-point dose-response curve for **1e**, **1e** with DMSO, **2e**, and **2e** with DMSO; Table S1: Simplified molecular input line entry (SMILE) for **1a**–**1e**; **2a**–**2e**; Table S2: Elemental analyis data for **1a**–**1e**; **2a**–**2e**; Table S3: Yields, melting points, and spectral assignments for **1a**–**1e**; **2a**–**2e**; Table S4: Crystal data and details of the structure determination of **1d**; Table S5: Optimized LC gradient used for purity and stability LC MS of **1a**–**1e**; **2a**–**2e**. This material is available free of charge via the Internet at pubs.acs.org.

## Abbreviations

Au(tht)Cl, tetrahydrothiophene gold(I) chloride; CCV, continuing calibration verification; CDCl_3_, deuterated chloroform; CL, confidence level; ESI-MS, electrospray ionization mass spectrometry; FBS, fetal bovine serum; FDA, Food and Drug Administration; FTIR, Fourier transform infrared; HCl, hydrochloric acid; HNO_3_, nitric acid; ICP-MS inductively coupled plasma mass spectrometry; IR, infrared spectroscopy; LC, liquid chromatography; MS, mass spectrometry; NMR, nuclear magnetic resonance; ppb, parts per billion; PPh_3_, triphenylphosphine; RA, rheumatoid arthritis TFP, (tri(2-furyl) phosphine; TrxR, thioredoxin reductase.
