# Supplementary material for: Evaluating the Antiproliferative Effects of Tri(2-Furyl)- and Triphenylphosphine-Gold(I) Pyridyl- and Pyrimidine-Thiolate Complexes
Source: Biomolecules. 2026 Jan 15;16(1):154. doi: 10.3390/biom16010154 (PMC12839414; doi:10.3390/biom16010154)
Supplement: Supplementary file 1 [file biomolecules-16-00154-s001.zip › biomolecules-4050997-supplementary-r2.pdf]

Supporting Information

Tri(2-furyl)- and Triphenylphosphine-Gold(I) Pyridyl- and Pyrimidine-Thiolate Complexes Exhibit Differential Antiproliferative Cytotoxic Activities

Kyle Wilhelm[1], Shyam Pokhrel[2], Drew Stolpman[1], Charli Worth[3], Sonal Mehta[1], Raul Villacob[1], Bernd Zechmann[4], Ahmad, Ahmad A. L.[2], Joseph Taube[3], Mitchell R. M. Bruce[2], Alice E. Bruce*[2], and Touradj Solouki*[1]*

[1] Department of Chemistry and Biochemistry, Baylor University, Waco, Texas

[2] Department of Chemistry, University of Maine, Orono, Maine

[3] Department of Biology, Baylor University, Waco, Texas

[4] Center for Microscopy and Imaging, Baylor University, Waco, Texas

KEYWORDS Auranofin, Cancer, Electrospray Ionization (ESI), Inductively coupled plasma mass spectrometry (ICP-MS), Gold Drugs

Figure S1(p. S2-S31). ^{1}H, ^{13}C, and ^{31}P NMR spectra for **1a-1e**; **2a-2e in $CDCl_3$**.

Figure S2 (p. S32-S33). ^{1}H, and ^{31}P NMR 72-hour spectra for **1e** and **2e**.

Figure S3 (p. S34-S43). Attenuated total reflectance (ATR)-FTIR spectra for **1a-1e**; **2a-2e**.

Figure S4 (p. S44). Calibration curve for ICP-MS analyses of gold concentrations.

Figure S5 (p. S45). UPLC-ESI-MS data for **1e** and **2e.**

Figure S6 (p. S46-S55). Positive-ion mode ESI-MS mass spectra for **1a-1e**; **2a-2e**.

Figure S7 (p. S563-S65). UV-Vis spectra for 1a-**1e** and 2a-**2e**.

Figure S8 (p. S66-S69). Eight-point dose-response curve for **1e**, **1e** with DMSO, **2e**, and **2e** with DMSO.

Table S1 (p. S70). Simplified molecular input line entry (SMILE) for **1a-1e**; **2a-2e**.

Table S2 (p. S71). Elemental analyis data for **1a-1e**; **2a-2e**.

Table S3 (p.S72-S73). Yields, melting points, and spectral assignments for **1a-1e**; **2a-2e**.

Table S4 (p. S74). Crystal data and details of the structure determination of **1d**

Table S5 (p. S75). Optimized LC gradient used for purity and stability LC MS of **1a-1e**; **2a-2e**.

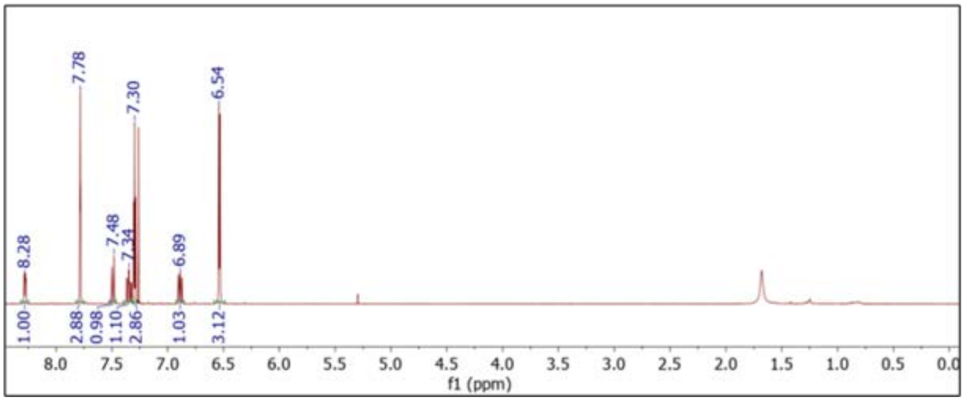

Figure S1A$_i$. ^{1}H NMR of (TFP)Au(Spy) compound **1a** in $CDCl_3$.

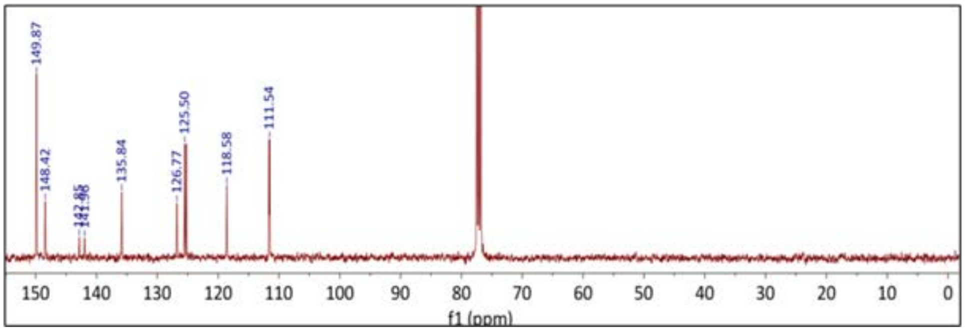

Figure S1A$_{\mathbf{ii}}$. ^{13}C NMR of (TFP)Au(Spy) **1a** in $CDCl_3$.

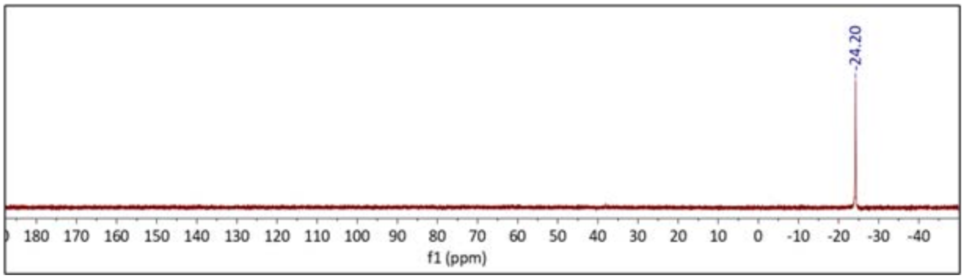

Figure S1A$_{iii}$. ^{31}P NMR of (TFP)Au(Spy) **1a** in $CDCl_3$.

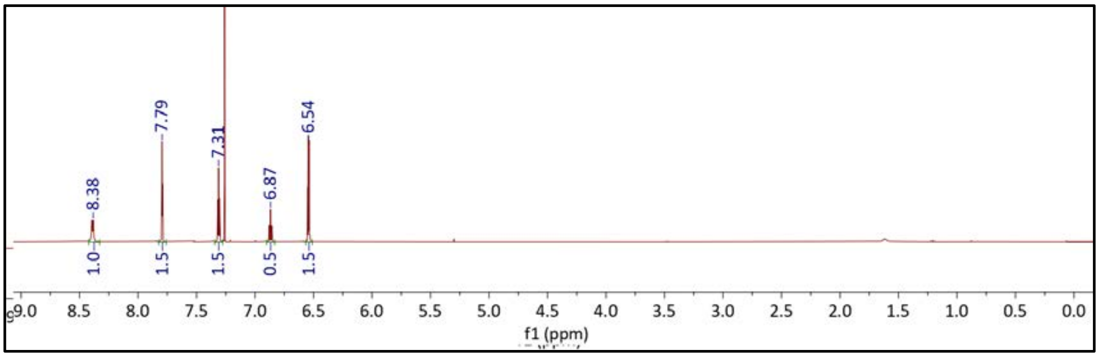

Figure S1B$_i$. ^{1}H NMR of (TFP)Au(Spyrim) **1b** in $CDCl_3$.

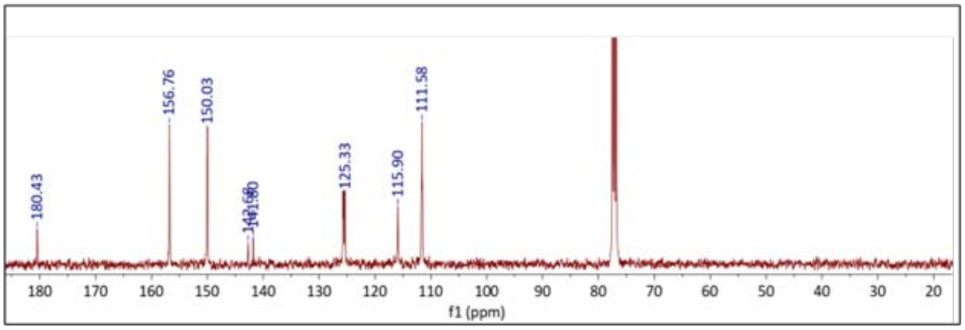

Figure S1B$_{ii}$. ^{13}C NMR of (TFP)Au(Spyrim) **1b** in $CDCl_3$.

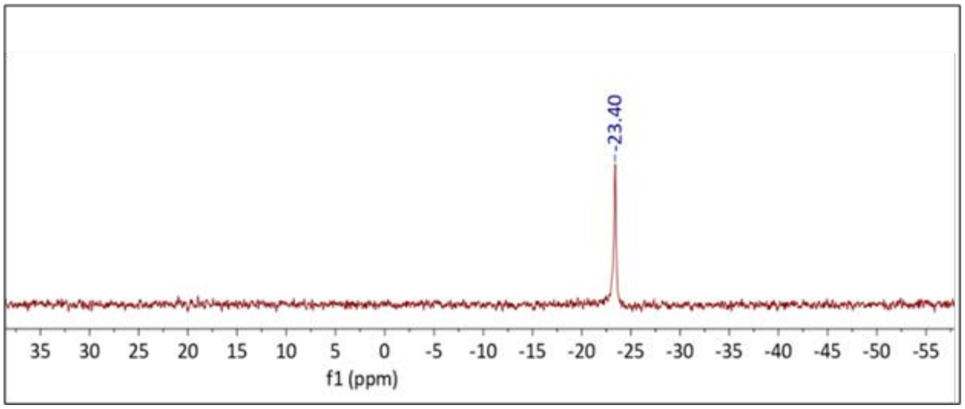

Figure S1B$_{iii}$. ^{31}P NMR of (TFP)Au(Spyrim) **1b** in $CDCl_3$.

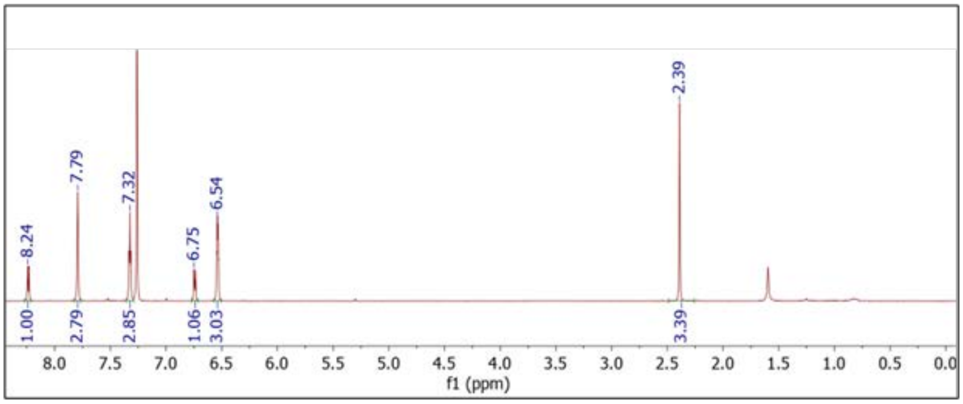

Figure S1C$_i$. ^{1}H NMR of (TFP)Au(SMepyrim) **1c** in $CDCl_3$.

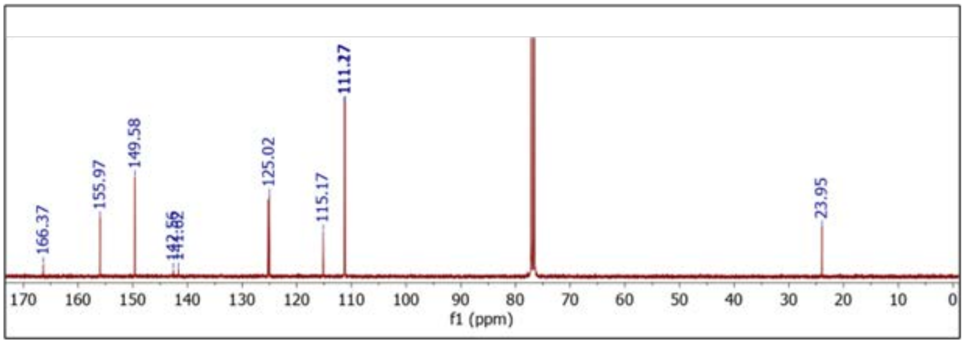

Figure S1C$_{ii}$. ^{13}C NMR of (TFP)Au(SMepyrim) **1c** in $CDCl_3$.

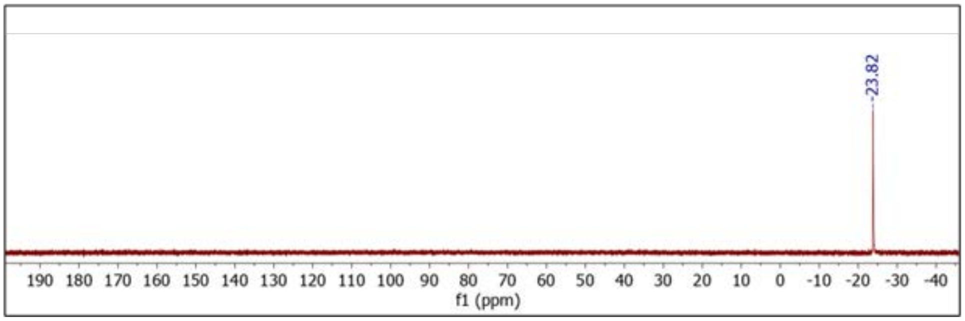

Figure S1C$_{iii}$. ^{31}P NMR of (TFP)Au(SMepyrim) **1c** in $CDCl_3$.

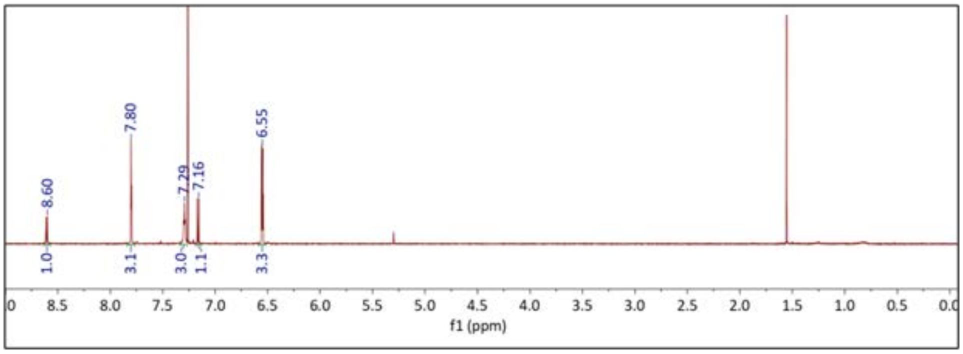

Figure S1D$_i$. ^{1}H NMR of (TFP)Au(SCF_3pyrim) **1d** in $CDCl_3$.

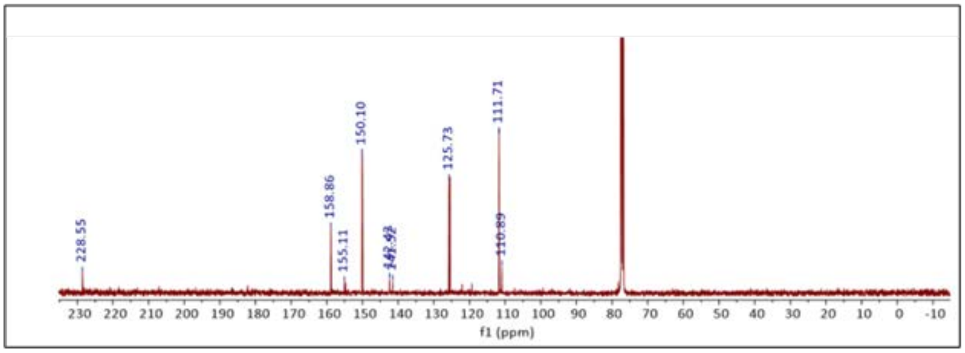

Figure S1D$_{ii}$. ^{13}C NMR of (TFP)Au(SCF$_3$pyrim) **1d** in $CDCl_3$.

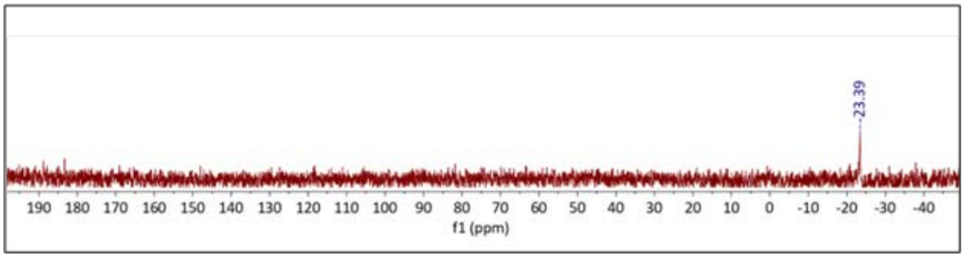

Figure S1D$_{iii}$. ^{31}P NMR of (TFP)Au(SCF_3pyrim) **1d** in $CDCl_3$.

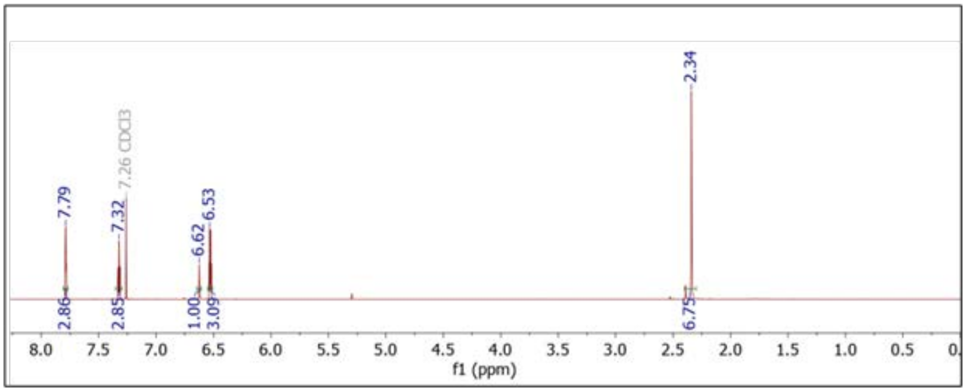

Figure S1E$_i$. ^{1}H NMR of (TFP)Au(SMe$_2$pyrim) **1e** in $CDCl_3$.

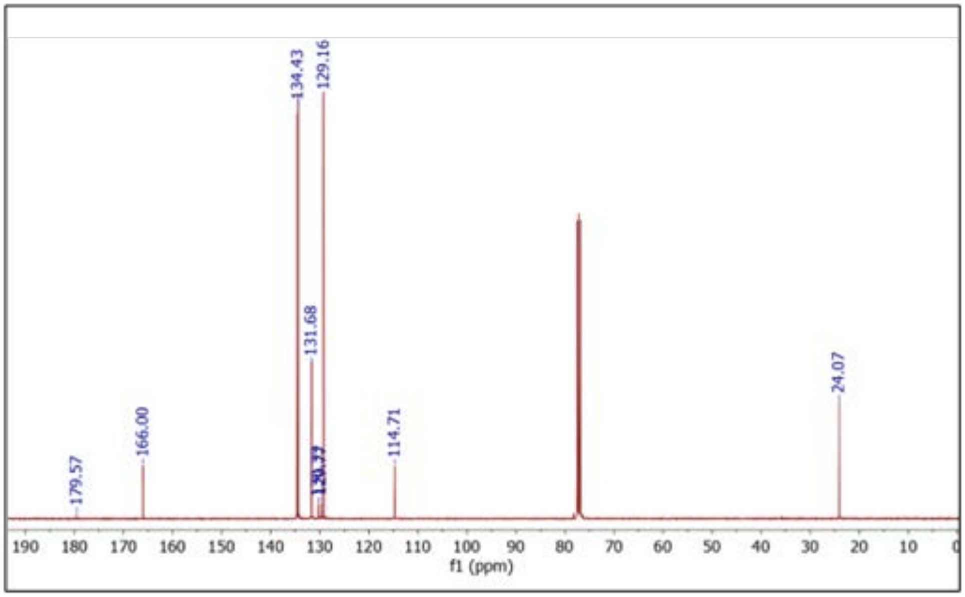

Figure S1E$_{ii}$. ^{13}C NMR of (TFP)Au(SMe$_2$pyrim) **1e** in $CDCl_3$.

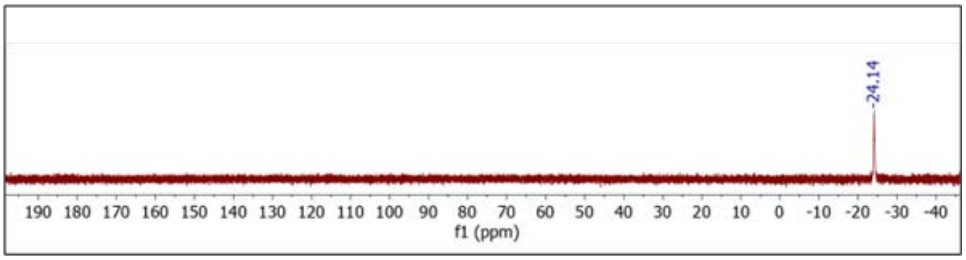

Figure S1E$_{iii}$. ^{31}P NMR of (TFP)Au(SMe$_2$pyrim) **1e** in $CDCl_3$.

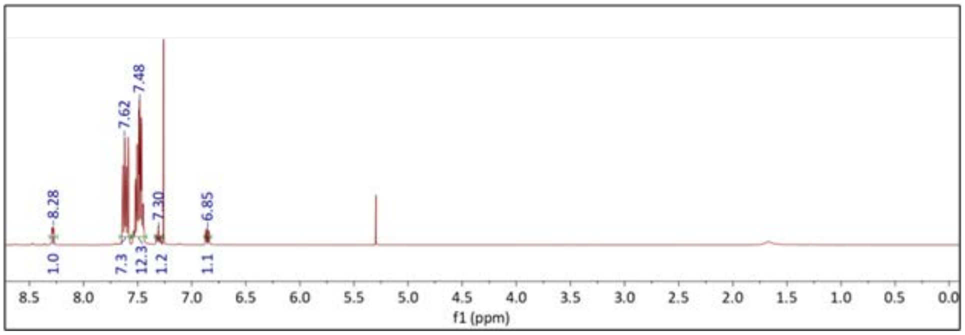

Figure S1F$_i$. ^{1}H NMR of $(Ph_3P)Au(Spy)$ **2a** in $CDCl_3$.

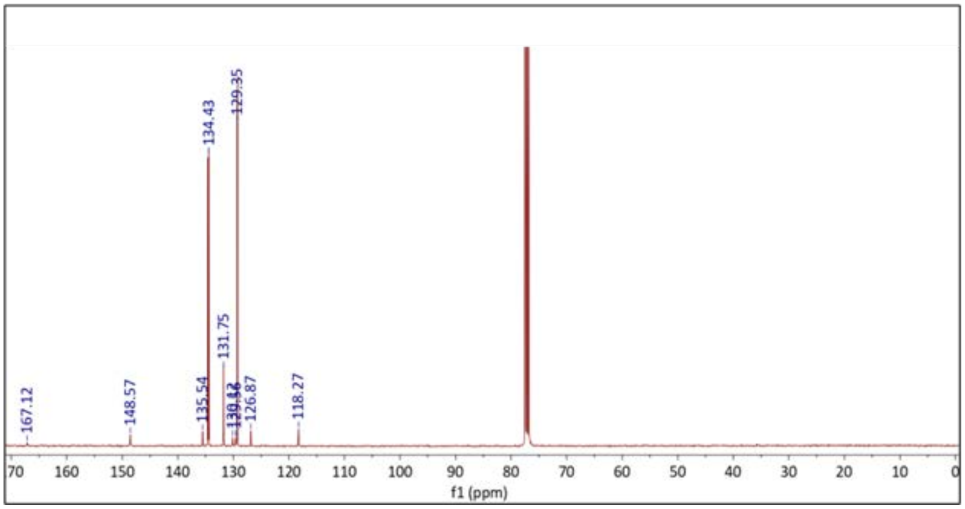

Figure S1F$_{ii}$. ^{13}C NMR of $(Ph_3P)Au(Spy)$ **2a** in $CDCl_3$.

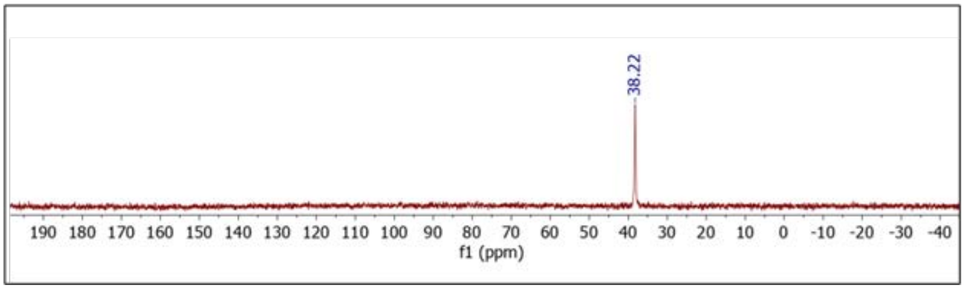

Figure S1F$_{iii}$. ^{31}P NMR of $(Ph_3P)Au(Spy)$ **2a** in $CDCl_3$.

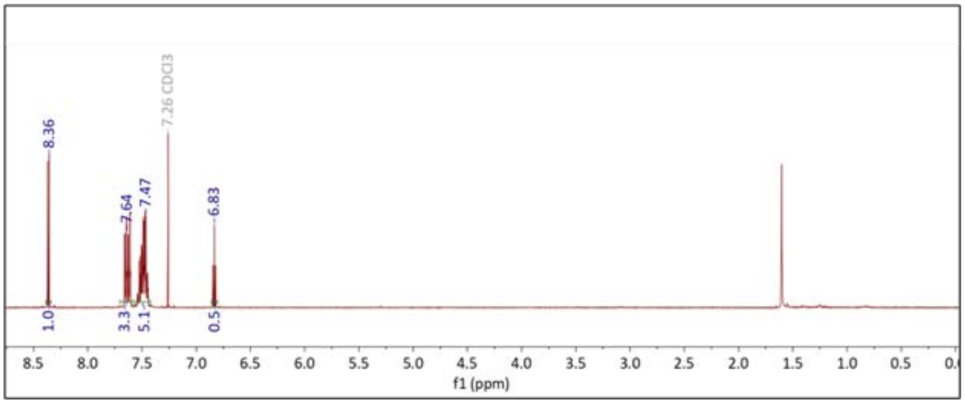

Figure S1Gi. ^{1}H NMR of $(Ph_3P)Au(Spyrim)$ **2b** in $CDCl_3$.

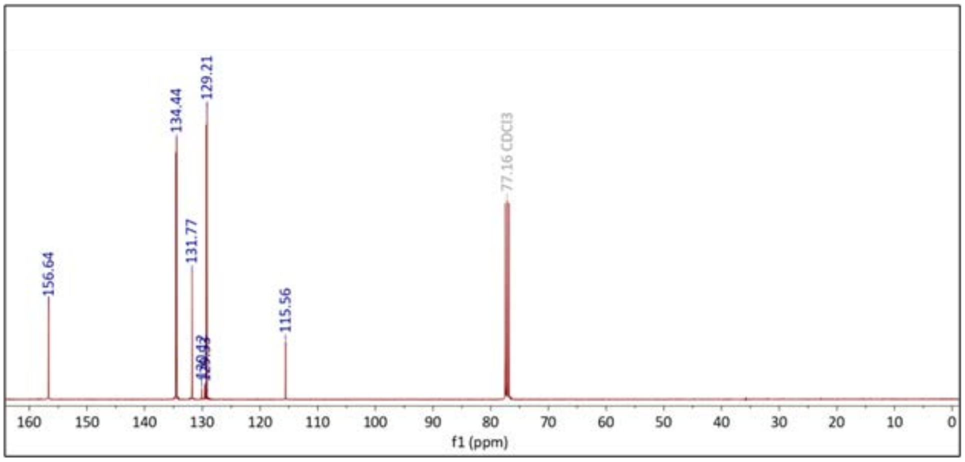

Figure S1G$_{ii}$. ^{13}C NMR of (Ph_3P)Au(Spyrim) **2b** in $CDCl_3$.

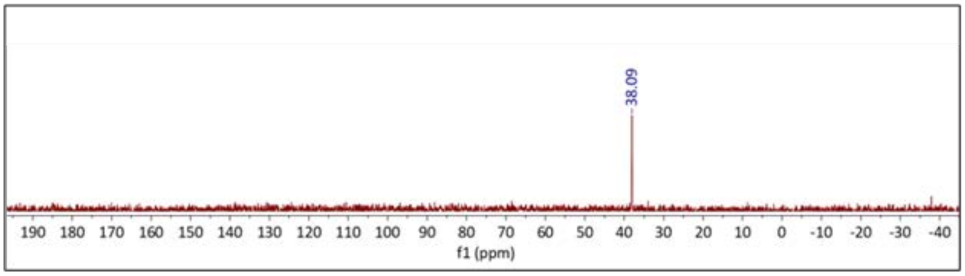

Figure S1G$_{iii}$. ^{31}P NMR of $(Ph_3P)Au(Spyrim)$ **2b** in $CDCl_3$.

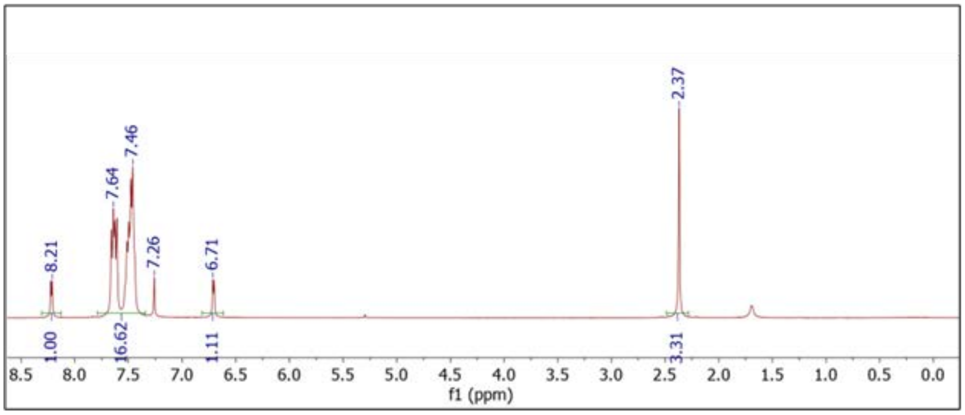

Figure S1Hi. ^{1}H NMR of $(Ph_3P)Au(SMepyrim)$ **2c** in $CDCl_3$.

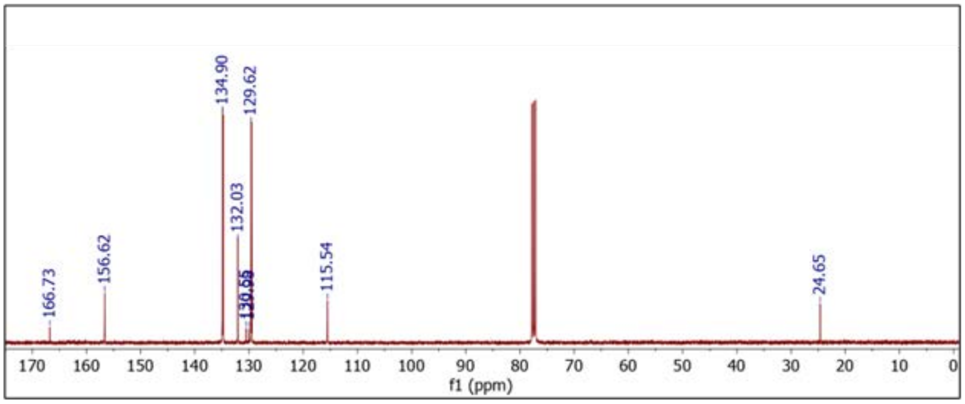

Figure S1H$_{ii}$. ^{13}C NMR of (Ph_3P)Au(SMepyrim) **2c** in $CDCl_3$.

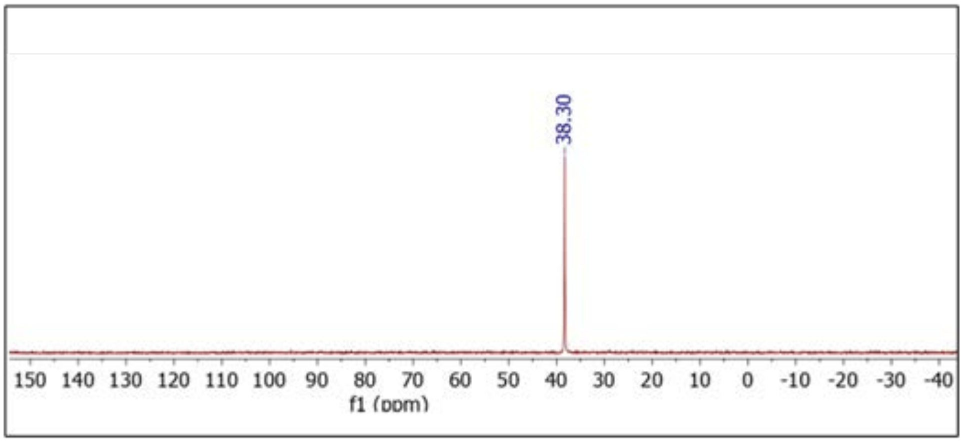

Figure S1H$_{iii}$. ^{31}P NMR of $(Ph_3P)Au(SMepyrim)$ **2c** in $CDCl_3$.

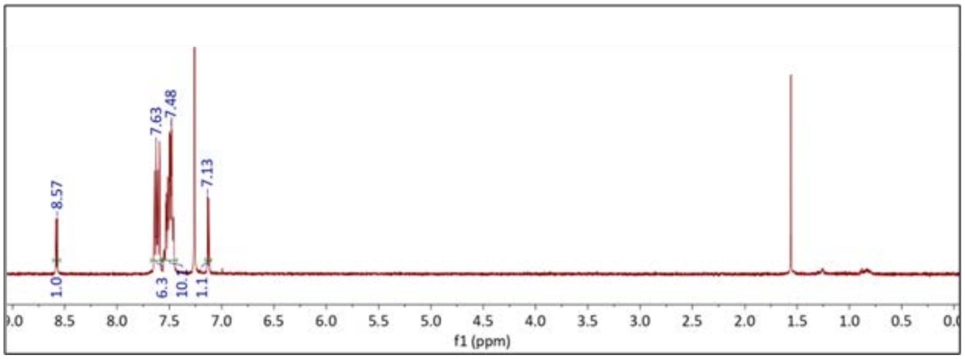

Figure S1I$_i$. ^{1}H NMR of $(Ph_3P)Au(SCF_3pyrim)$ **2d** in $CDCl_3$.

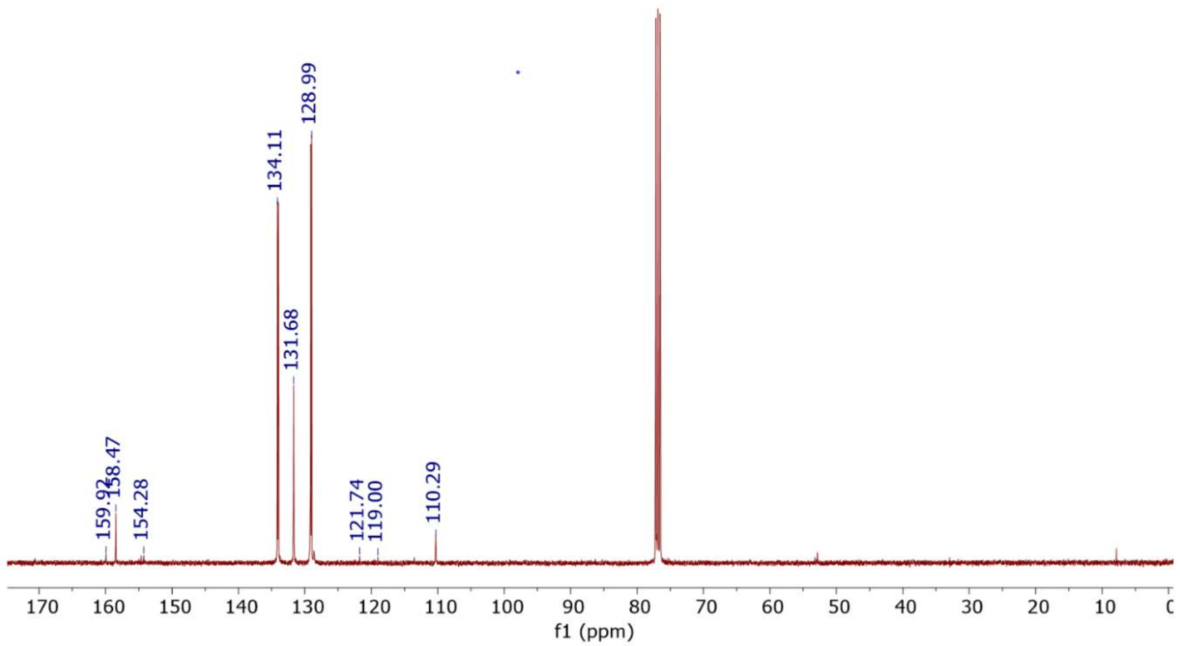

Figure S1I$_{ii}$. ^{13}C NMR of $(Ph_3P)Au(SCF_3pyrim)$ **2d** in $CDCl_3$.

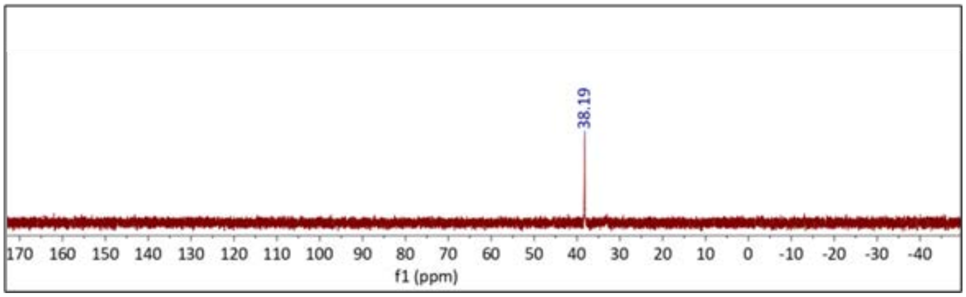

Figure S1I$_{iii}$. ^{31}P NMR of (Ph_3P)Au(SCF_3pyrim) **2d** in $CDCl_3$.

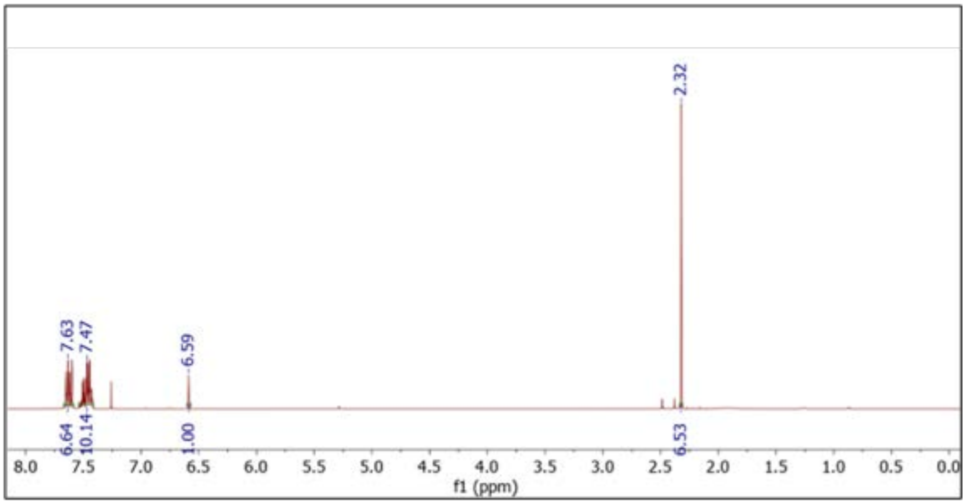

Figure S1J$_i$. ^{1}H NMR of $(Ph_3P)Au(SMe_2pyrim)$ **2e** in $CDCl_3$.

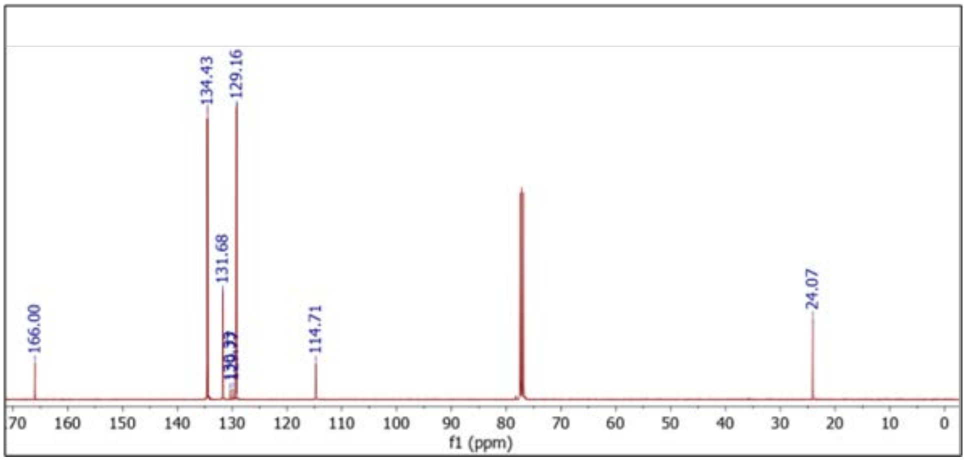

Figure S1J$_{ii}$. ^{13}C NMR of $(Ph_3P)Au(SMe_2pyrim)$ **2e** in $CDCl_3$.

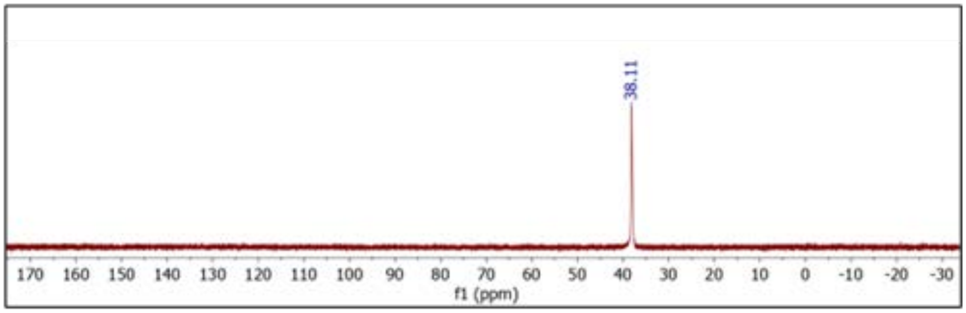

Figure S1J$_{iii}$. ^{31}P NMR of $(Ph_3P)Au(SMe_2pyrim)$ **2e** in $CDCl_3$.

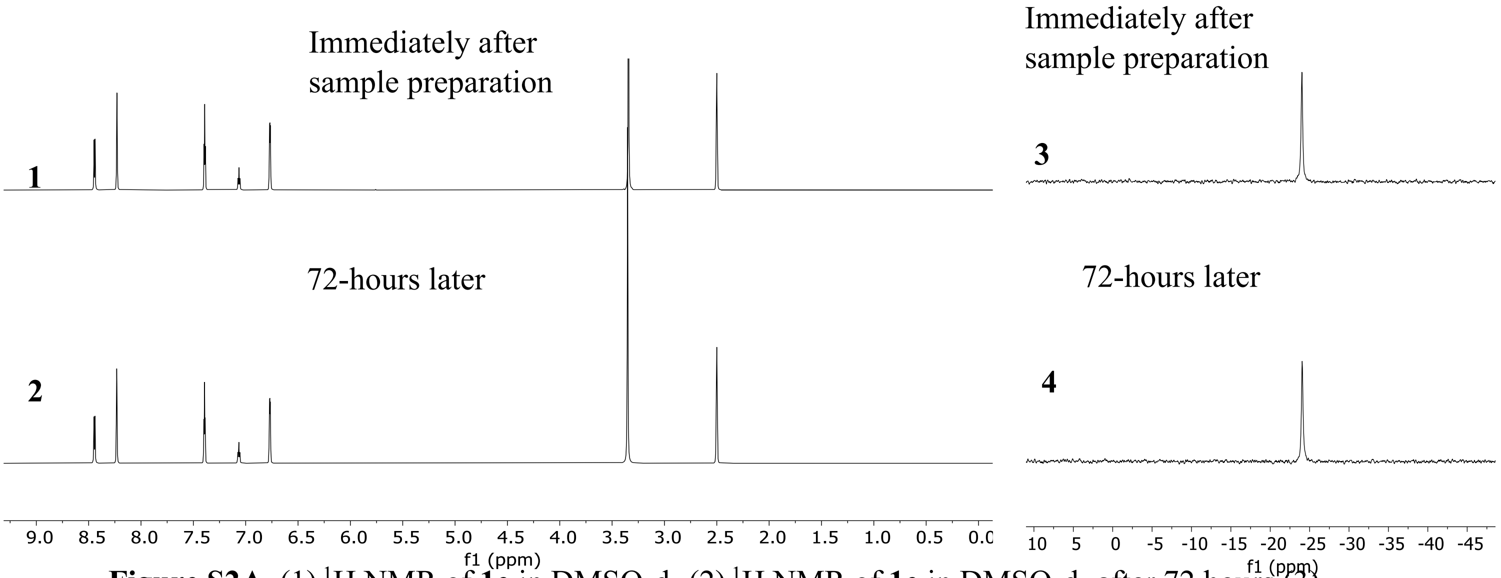

Figure S2A. (1) ^{1}H NMR of **1e** in DMSO-d_6 (2) ^{1}H NMR of **1e** in DMSO-d_6 after 72 hours (3) ^{31}P{^{1}H} NMR of **1e** in DMSO-d_6, and (4) ^{31}P{^{1}H} NMR of **1e** in DMSO-d_6 after 72 hours.

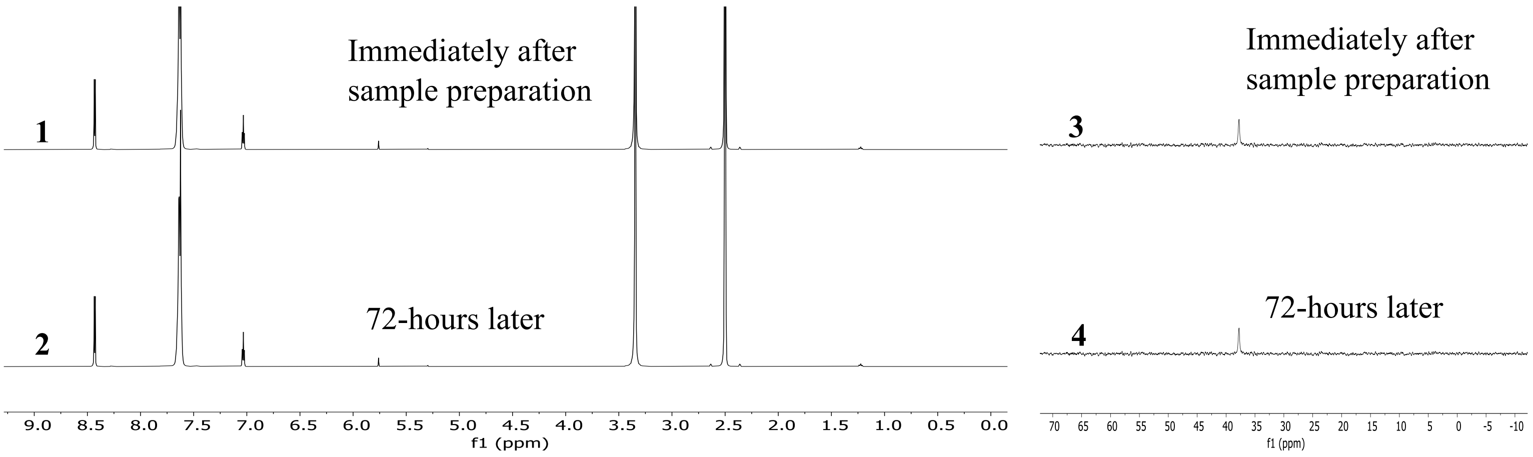

Figure S2B. (1) ^{1}H NMR of 2**e** in DMSO-d_6, (2) ^{1}H NMR of **2e** in DMSO-d_6 after 72h hours, (3) ^{31}P{^{1}H} NMR of 2**e** in DMSO-d_6, and (4) ^{31}P{^{1}H} NMR of **2e** in DMSO-d_6 after 72 hours.

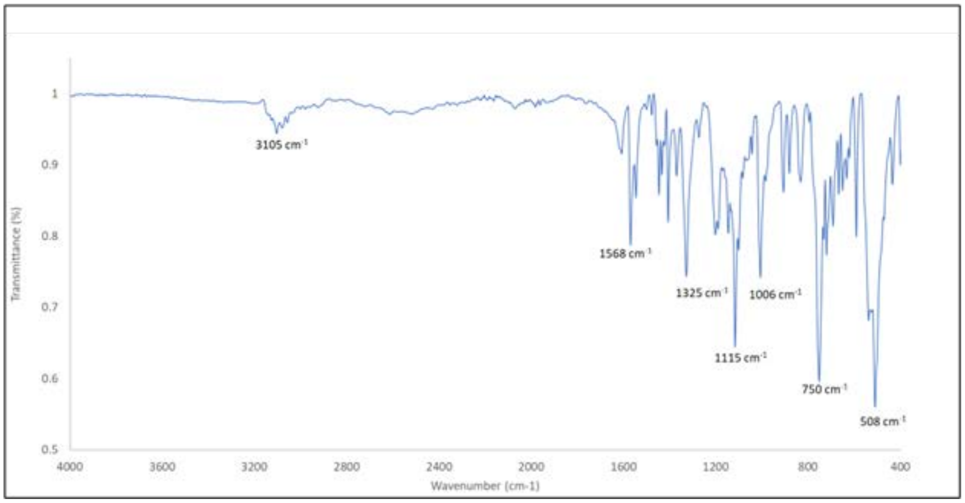

Figure S3A. ATR-FTIR of (TFP)Au(Spy) **1a**.

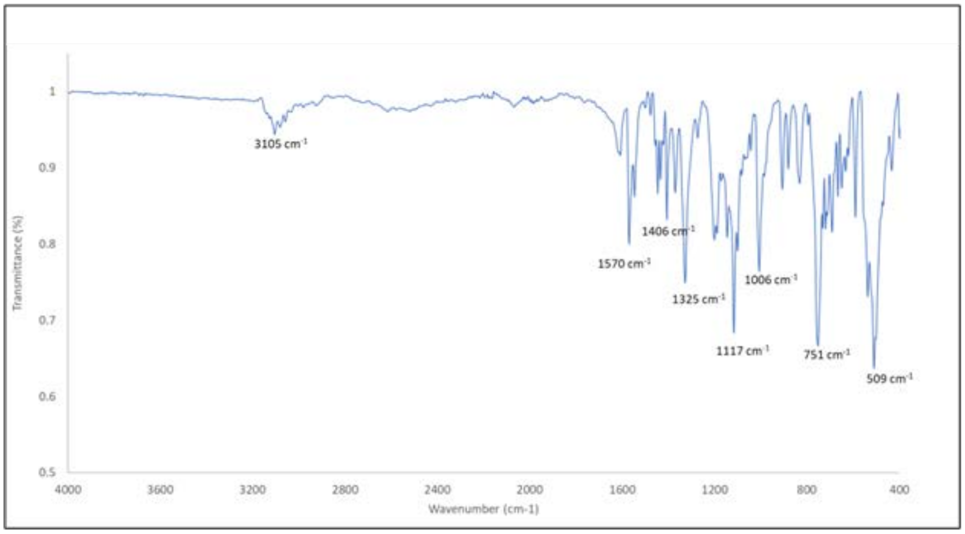

Figure S3B. ATR-FTIR of (TFP)Au(Spyrim) **1b**.

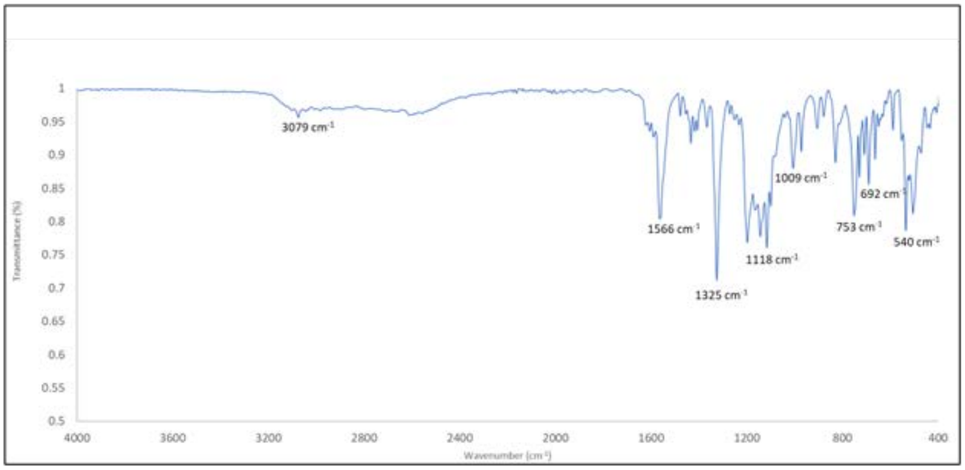

Figure S3C. ATR-FTIR of (TFP)Au(SMepyrim) **1c**.

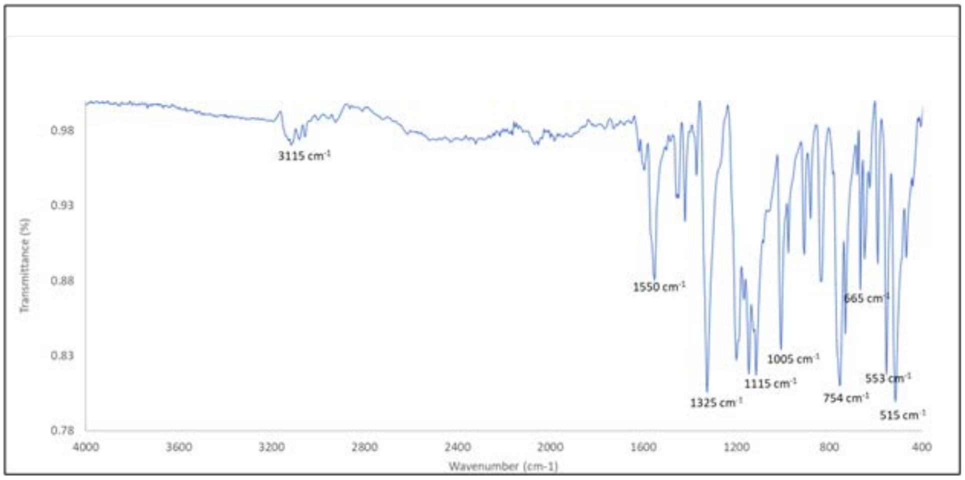

Figure S3D. ATR-FTIR of (TFP)Au(SCF_3pyrim) **1d**.

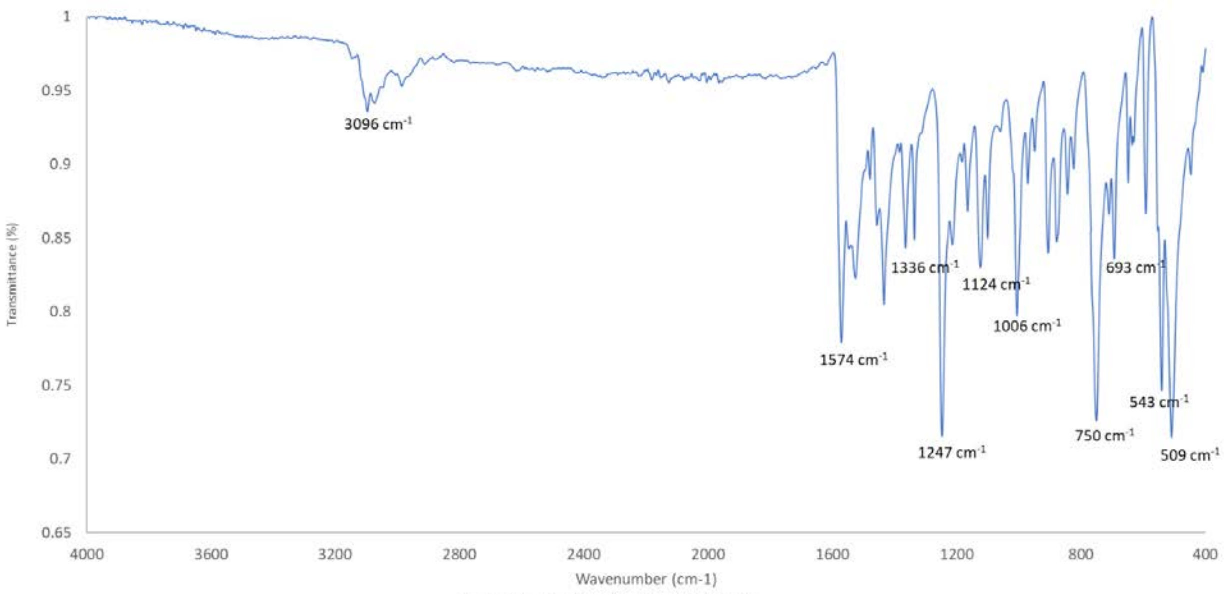

Figure S3E. ATR-FTIR of (TFP)Au(SMe$_2$pyrim) **1e**.

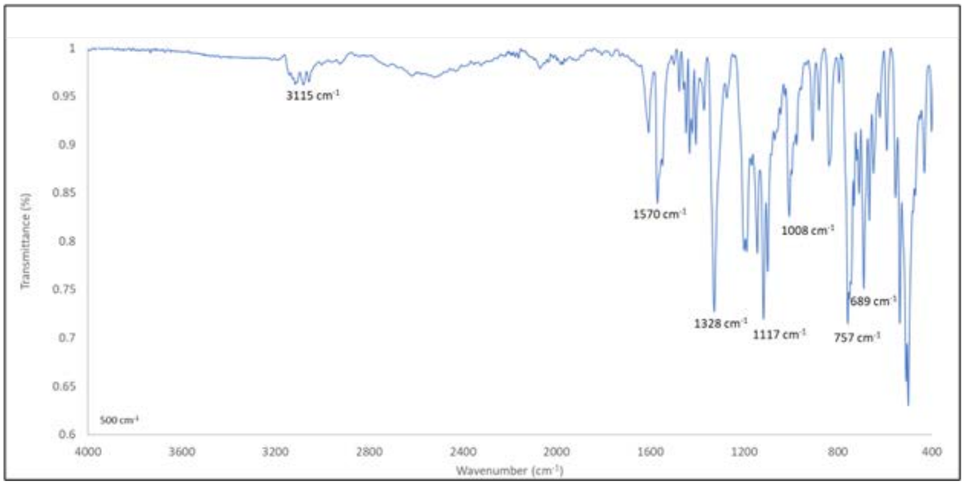

Figure S3F. ATR-FTIR of $(Ph_3P)Au(Spy)$ **2a**.

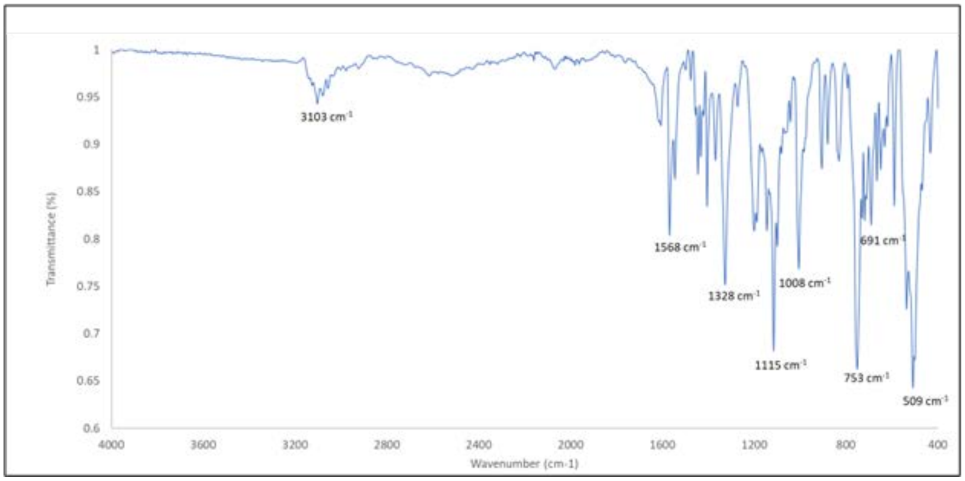

Figure S3G. ATR-FTIR of $(Ph_3P)Au(Spyrim)$ **2b**.

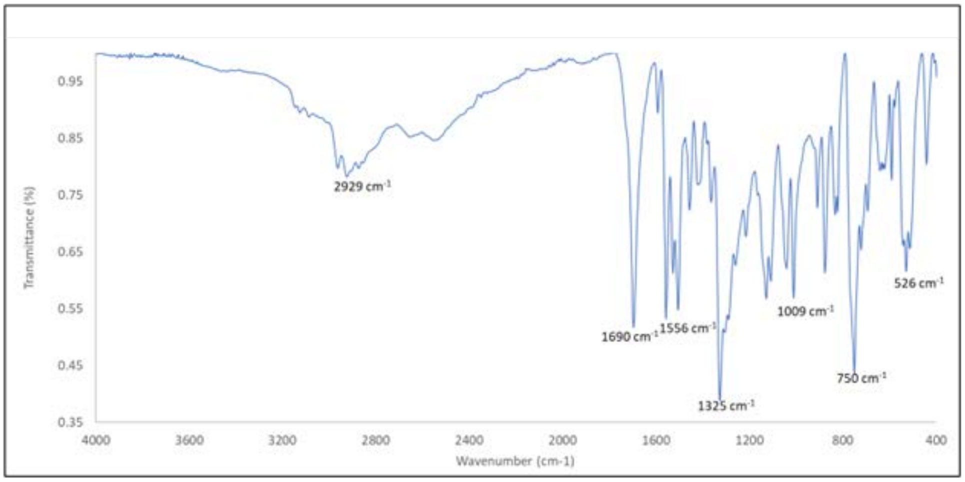

Figure S3H. ATR-FTIR of $(Ph_3P)Au(SMepyrim)$ **2c**.

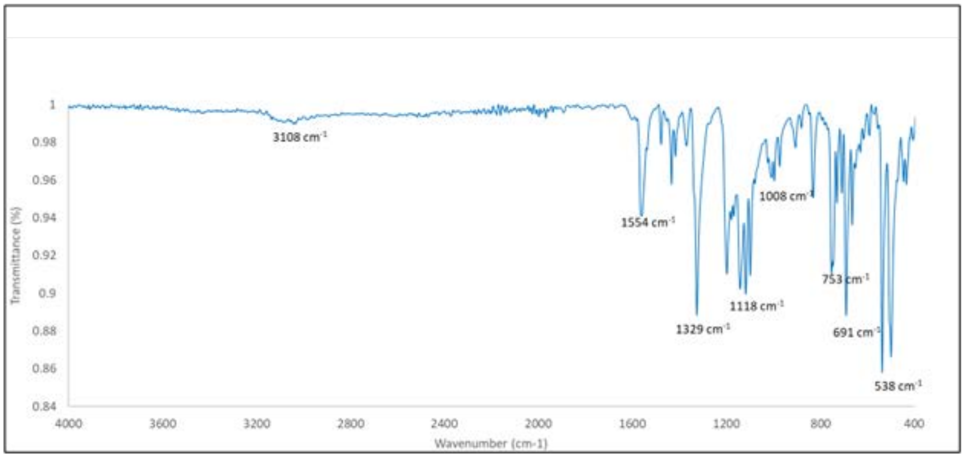

Figure S3I. ATR-FTIR of $(Ph_3P)Au(SCF_3pyrim)$ **2d**.

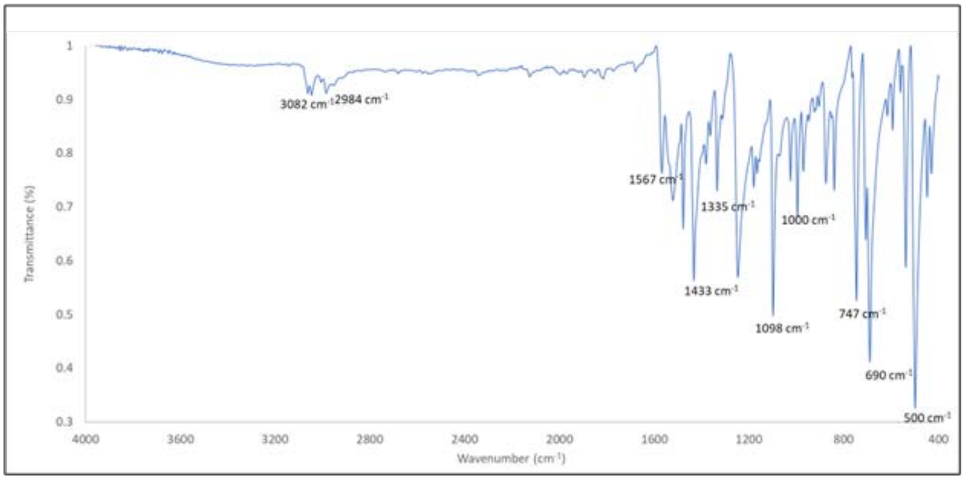

Figure S3J. ATR-FTIR of $(Ph_3P)Au(SMe_2pyrim)$ **2e**.

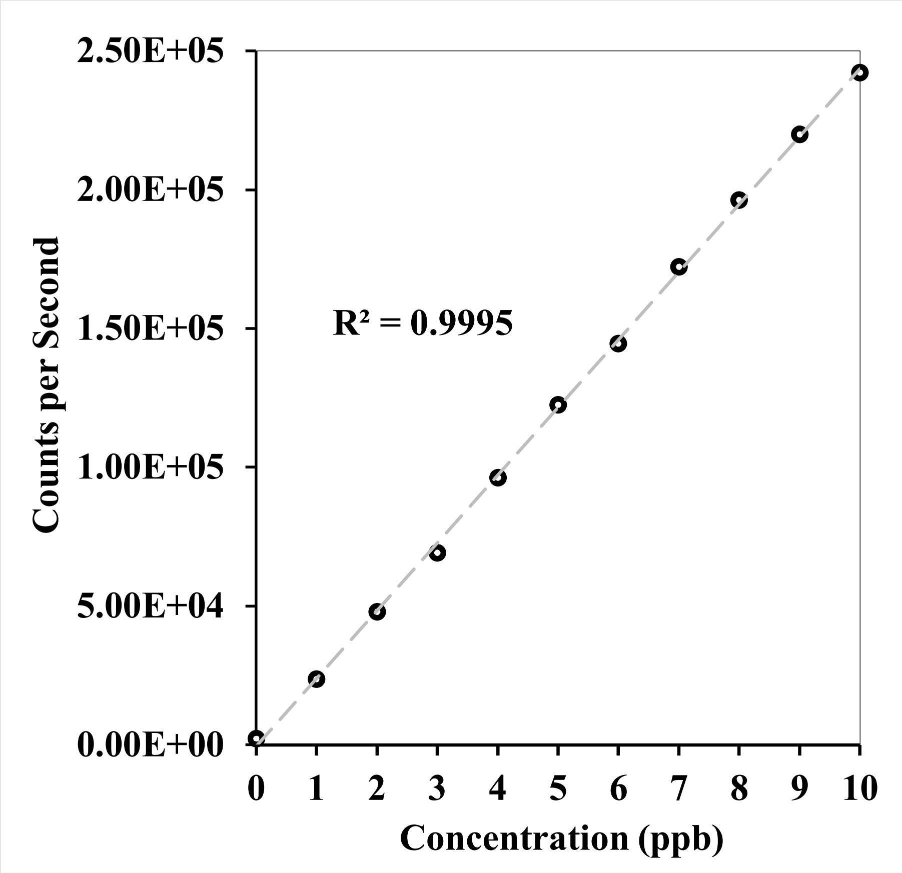

Figure S4. The Au ICP-MS external calibration curve for ICP-MS experiment to determine the solubilities of all the complexes (**1a-2e**) and auranofin in cell media. Each point is represented at the 95% confidence interval (n = 48); based on the replicate ICP-MS measurements for determination of gold concentrations, calculated relative uncertainties with error propagations included (as represented by the width and height of each calibration point in this plot), were less than 2% for all analyzed samples.

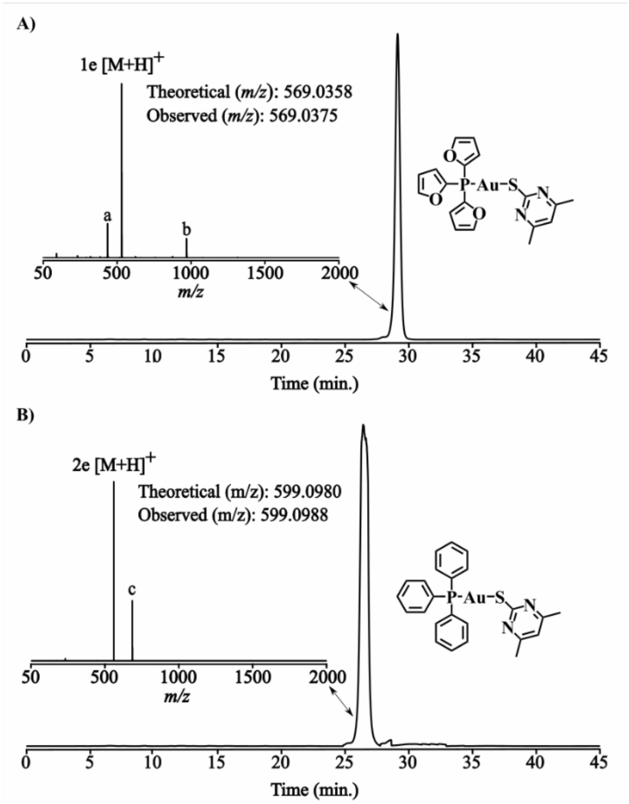

Figure S5. The LC-MS total ion chromatograms (for *m/z* range 50 to 2000) for the retention time (RT) ranges of 0 to 45 minutes for gold(I) complexes **1e** (top panel, **A**) and **2e** (bottom panel, **B**) indicate elution of single complexes at RT 29.12 minutes and RT 26.05 minutes, respectively. The protonated pseudo molecular ions ($[M+H]^+$) were the most abundant species observed in the respective mass spectra for **1e** (inset of top panel, **A**) and **2e** (inset of bottom panel, **B**). Two ion types labeled as "a" and "b" were observed at *m/z* values of 470.0657, 969.0044 for **1e** and another labeled "c" at *m/z* 660.0872 for **2e**. These ions are presumed to be products of gas-phase ion-molecule reactions as they only appear during the LC elution of complexes **1e** or **2e**. Based on the experimental mass measurement accuracy of better than 5 ppm, potential chemical compositions of the ions labeled "a", "b", and "c', can be assigned as $[(TFP)AuSC]^+$ ($C_{16}H_{18}AuPS$), $[(TFP)Au]_2Spyrim]^+$ ($C_{29}H_{22}Au_2NO_6P_2S$), and $[(PPh_3)_2Au]^+$ ($C_{16}H_{18}AuPS$), respectively. These mass spectrometry data interpretations (for product ion formations in the gas phase) are further validated with ^{31}P NMR results which confirm the purity of the precursors (complexes **1e** and **2e**) as well as their stability over the 72-hour testing period (see **Figure S2**)."

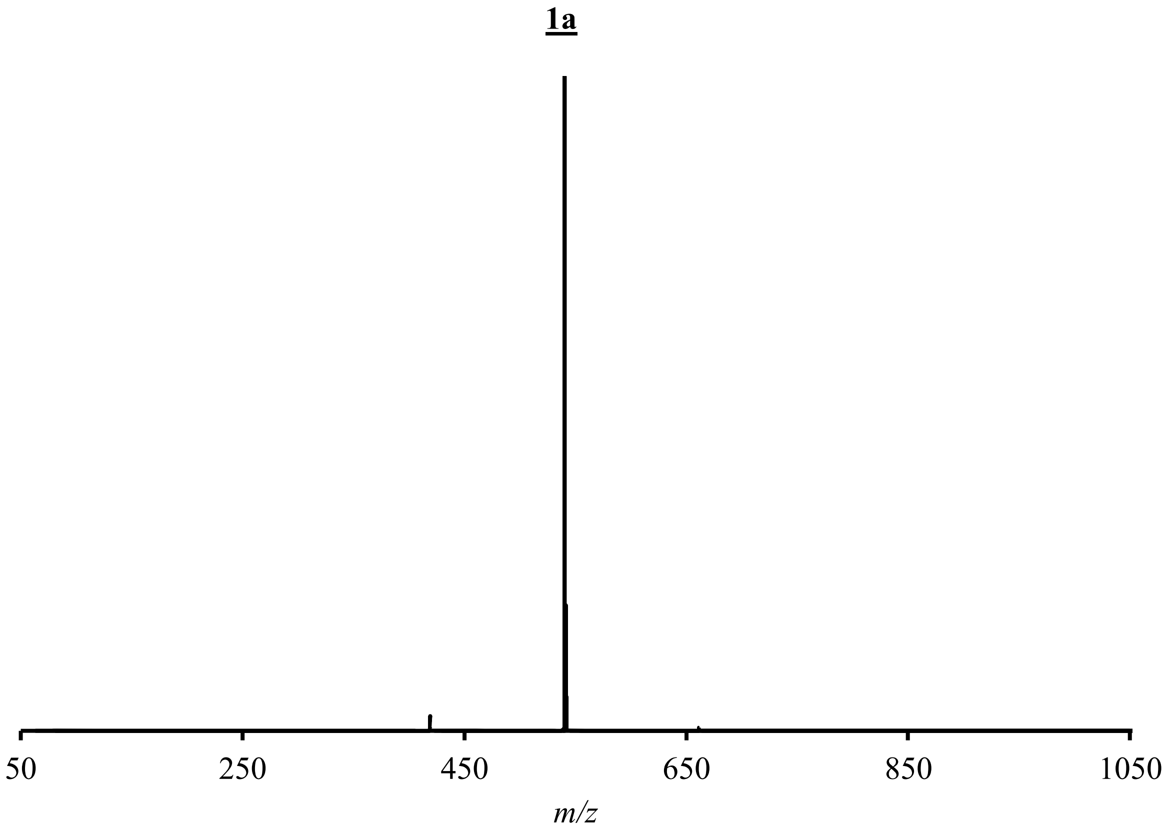

Figure S6A. Positive-ion mode ESI of (TFP)Au(Spy) **1a**.

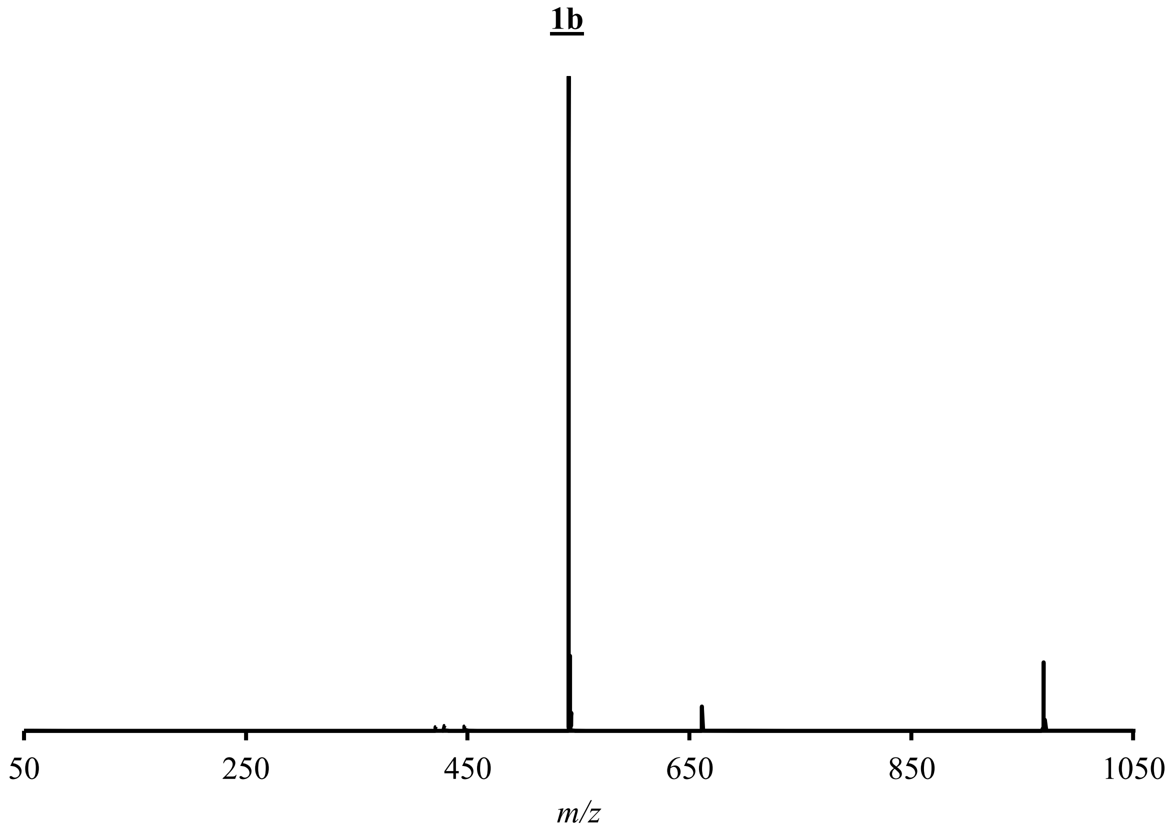

Figure S6B. Positive-ion mode ESI-MS of (TFP)Au(Spyrim) **1b**.

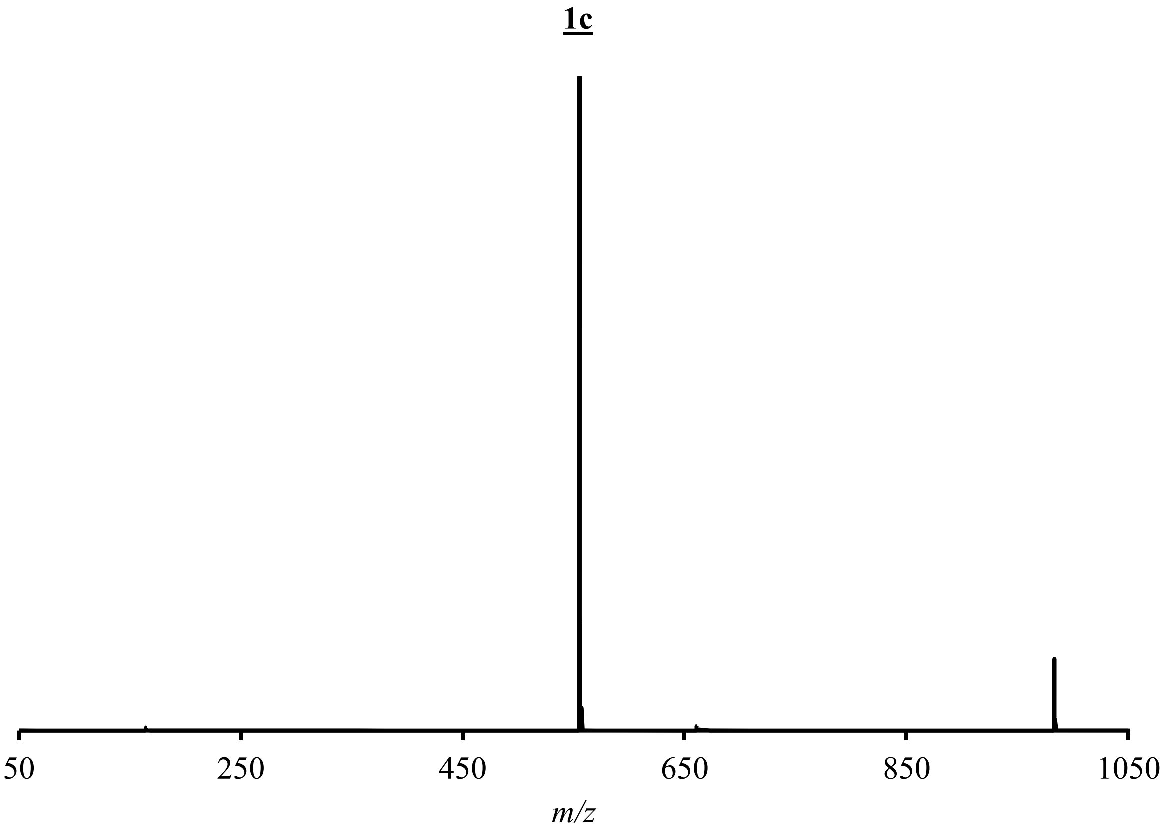

Figure S6C. Positive-ion mode ESI-MS of (TFP)Au(SMepyrim) **1c**.

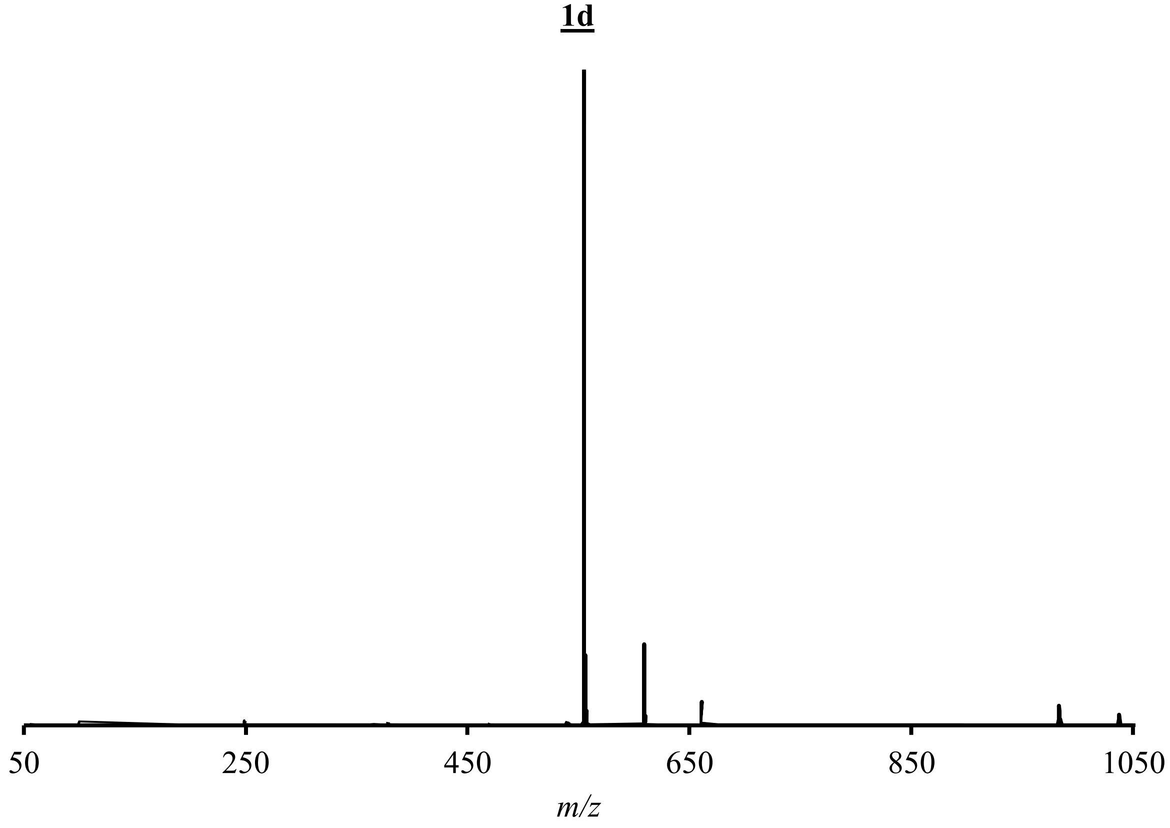

Figure S6D. Positive-ion mode ESI-MS of (TFP)Au(SCF_3pyrim) **1d**.

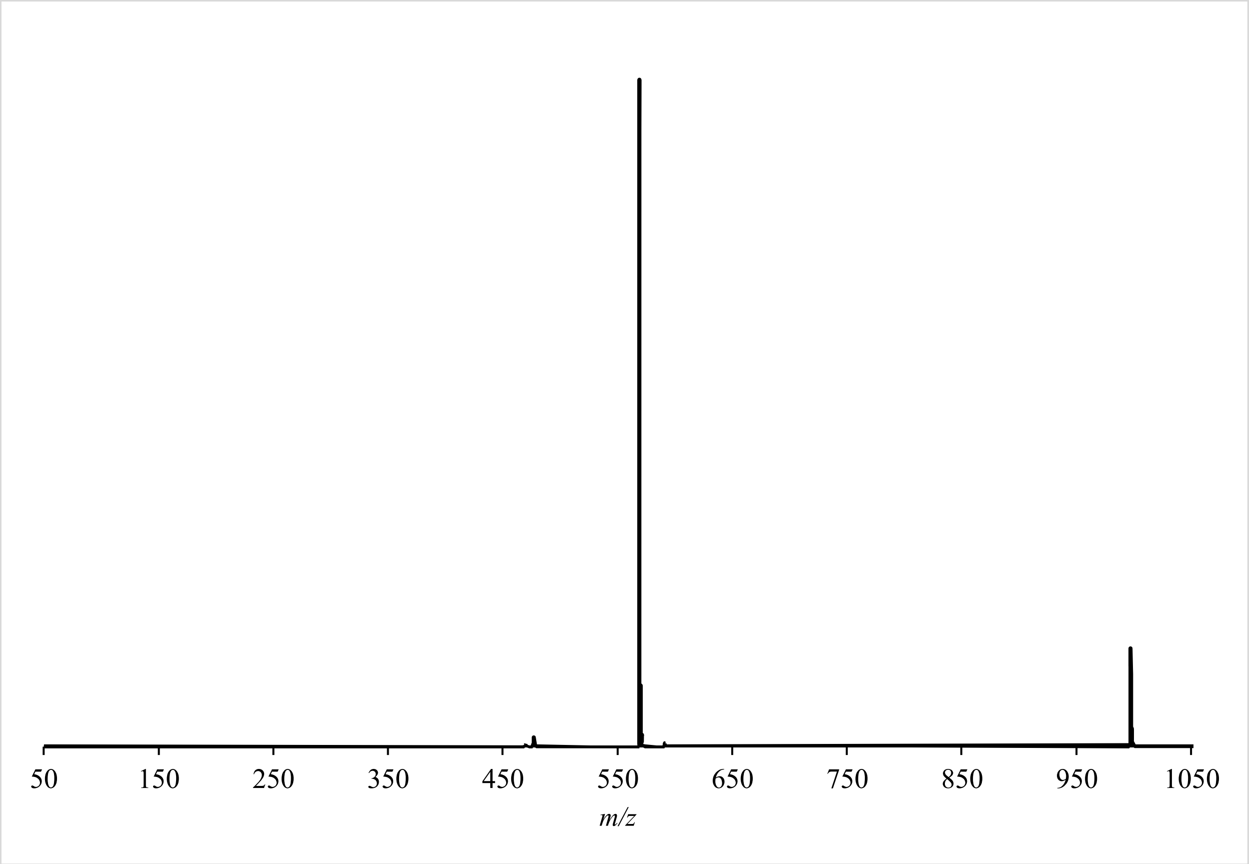

Figure S6E. Positive-ion mode ESI-MS of (TFP)Au(SMe$_2$pyrim) **1e**.

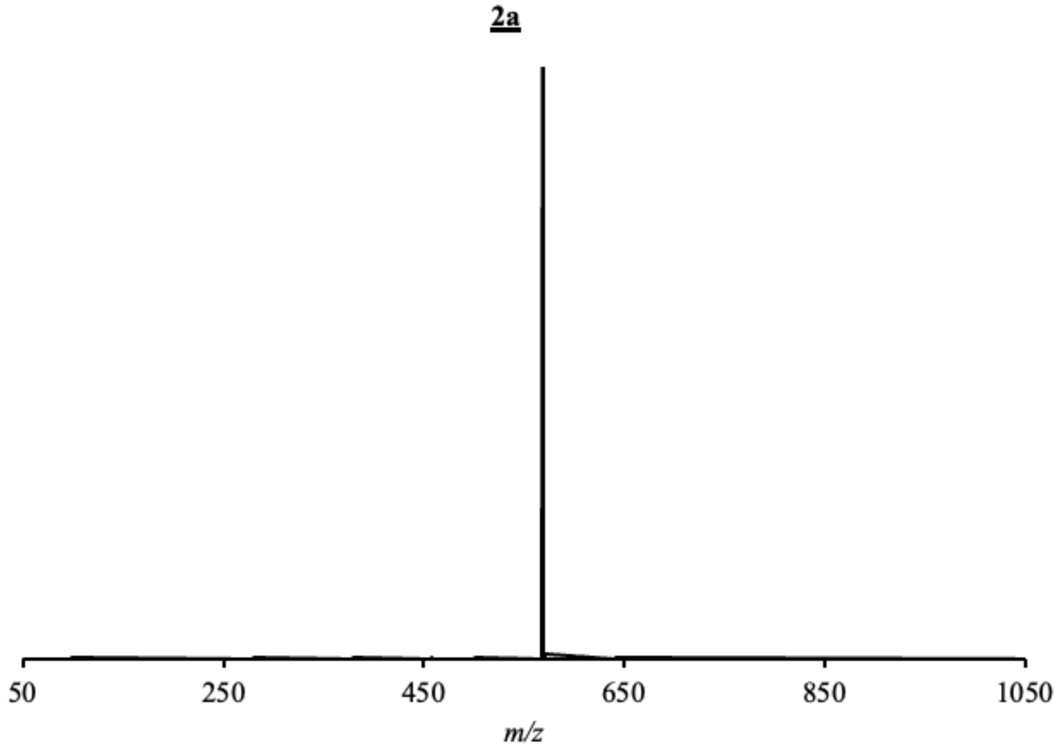

Figure S6F. Positive-ion mode ESI-MS of (Ph3P)Au(Spy) **2a**.

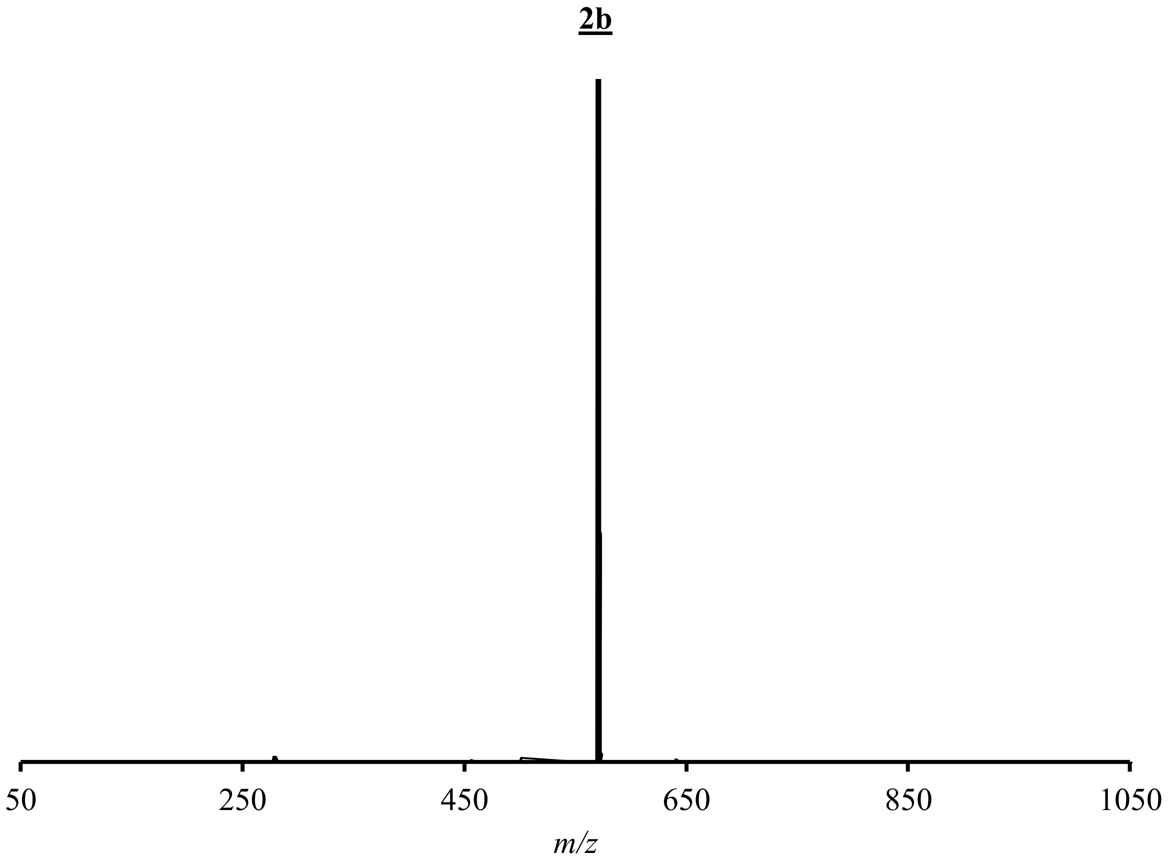

Figure S6G. Positive-ion mode ESI-MS of (Ph3P)Au(Spyrim) **2b**

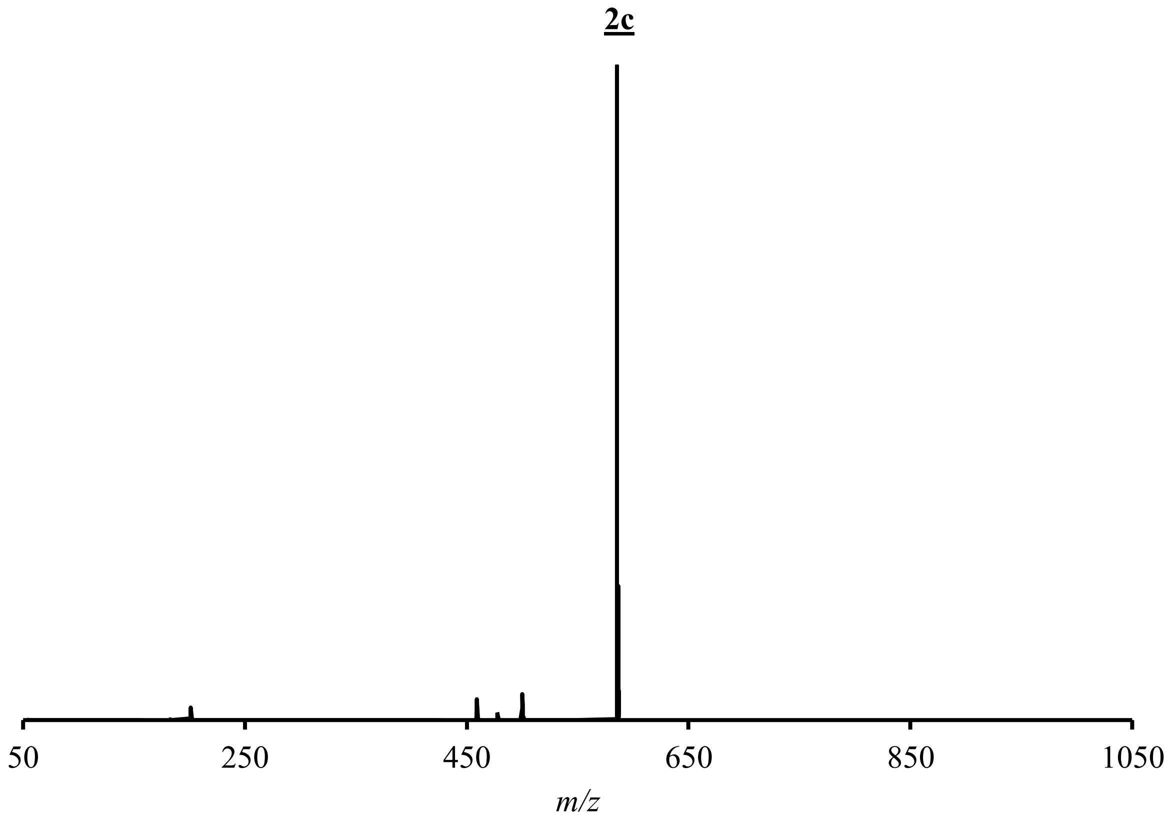

Figure S6H. Positive-ion mode ESI-MS of (Ph3P)Au(SMepyrim) **2c**.

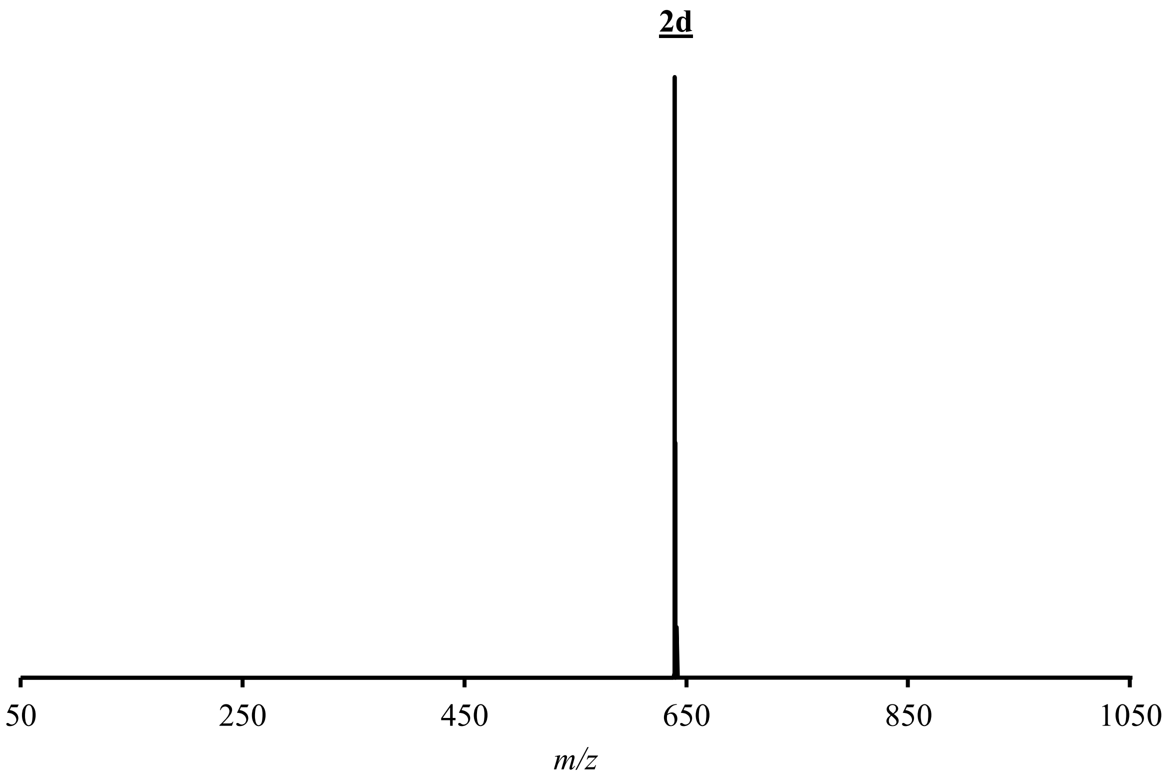

Figure S6I. Positive-ion mode ESI-MS of (Ph3P)Au(SCF_3pyrim) **2d**

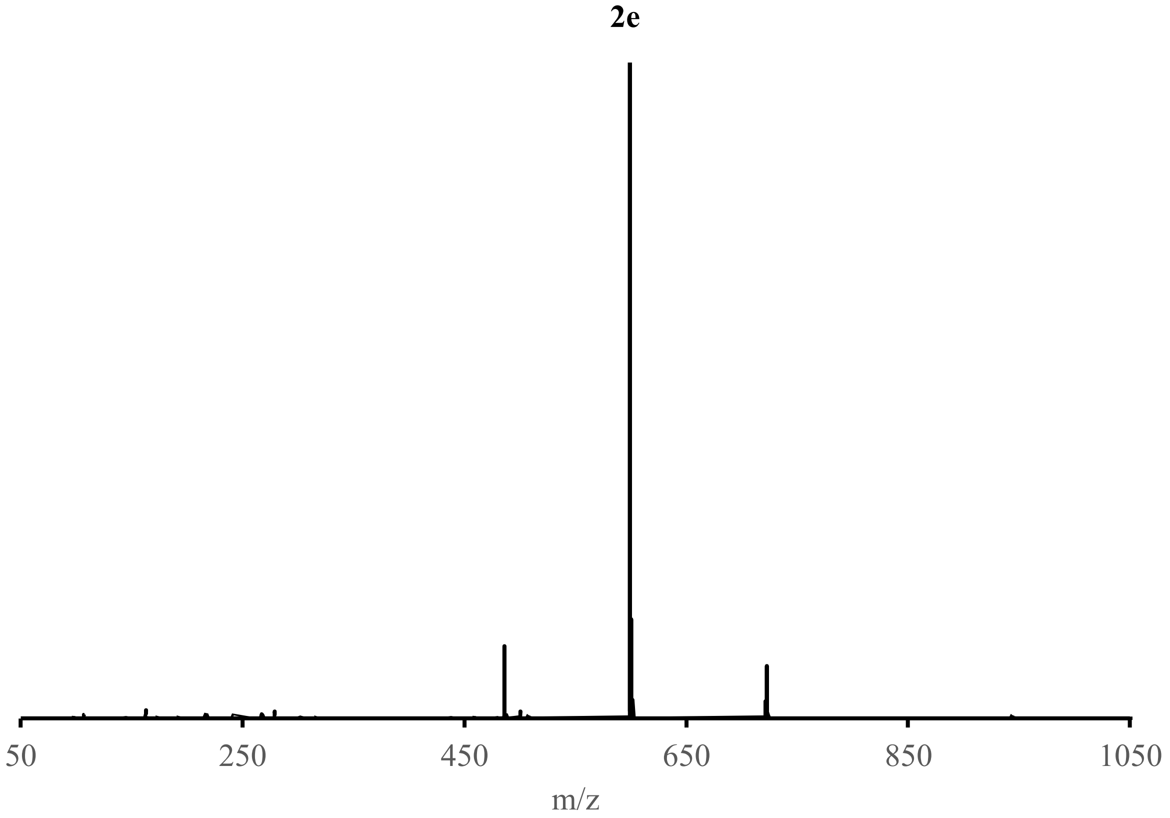

Figure S6J. Positive-ion mode ESI-MS of (Ph3P)Au(SMe2pyrim) **2e**.

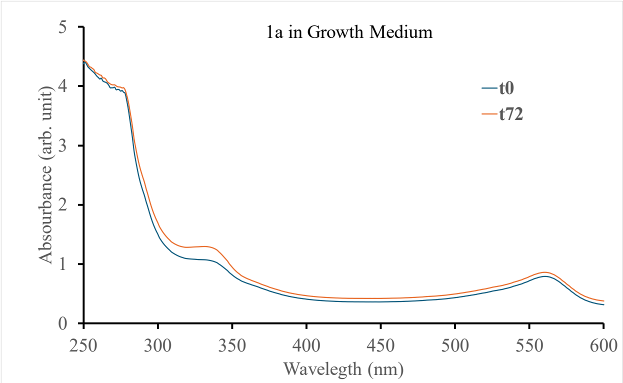

Figure S7A. UV-Vis Spectra of compound **1a** dissolved in cell media with 1% (v/v) DMSO immediately after sample preparation (blue trace, “T0”) and after 72 hours (yellow trace, “T72”); no observable changes were noticed between the two runs.

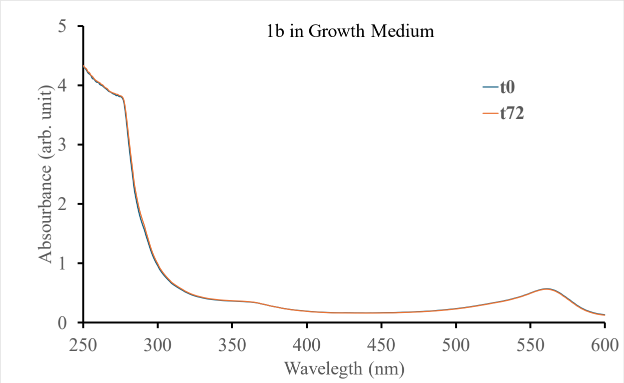

Figure S7B. UV-Vis Spectra of compound **1b** dissolved in cell media with 1% (v/v) DMSO immediately after sample preparation (blue trace, “T0”) and after 72 hours (yellow trace, “T72”); no observable changes were noticed between the two runs.

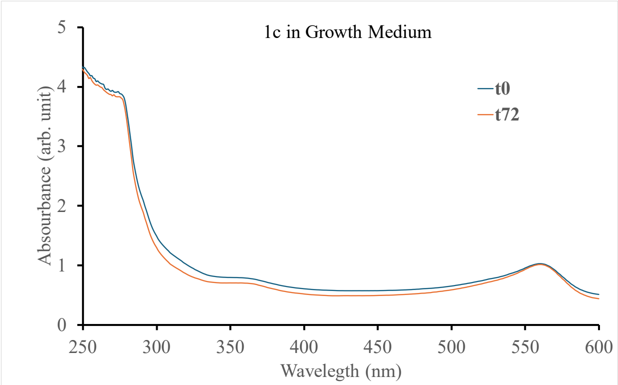

Figure S7C. UV-Vis Spectra of compound **1c** dissolved in cell media with 1% (v/v) DMSO immediately after sample preparation (blue trace, "T0") and after 72 hours (yellow trace, "T72"); no observable changes were noticed between the two runs.

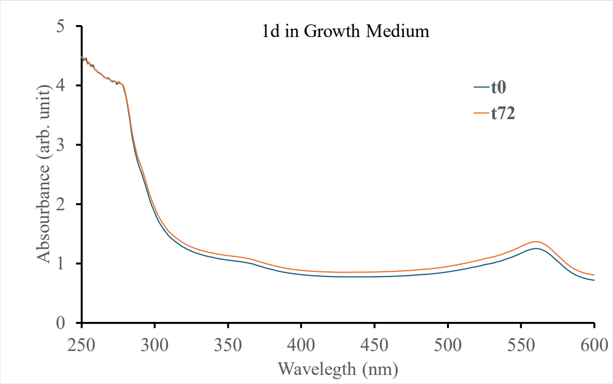

Figure S7D. UV-Vis Spectra of compound **1d** dissolved in cell media with 1% (v/v) DMSO immediately after sample preparation (blue trace, “T0”) and after 72 hours (yellow trace, “T72”); no observable changes were noticed between the two runs.

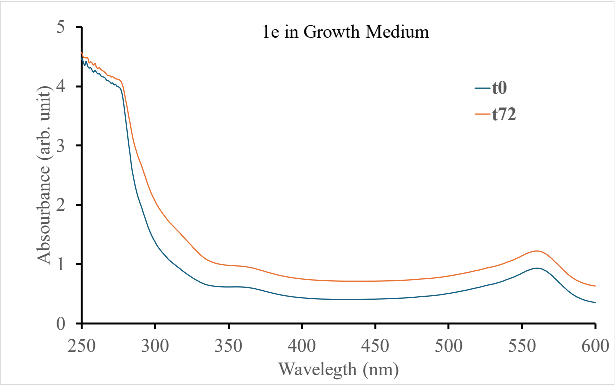

Figure S7E. UV-Vis Spectra of compound **1e** dissolved in cell media with 1% (v/v) DMSO immediately after sample preparation (blue trace, “T0”) and after 72 hours (yellow trace, “T72”); no observable changes are noticed between the two runs.

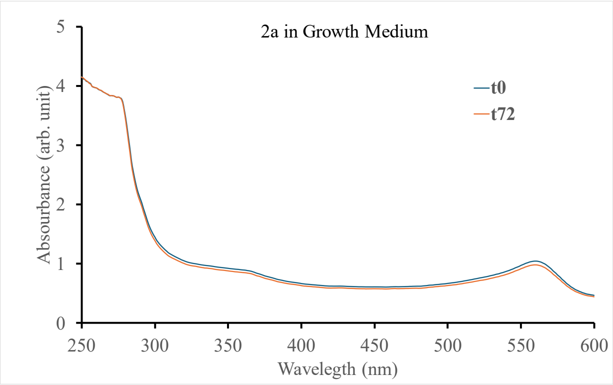

Figure S7F. UV-Vis Spectra of compound **2a** dissolved in cell media with 1% (v/v) DMSO immediately after sample preparation (blue trace, "T0") and after 72 hours (yellow trace, "T72"); no observable changes were noticed between the two runs.

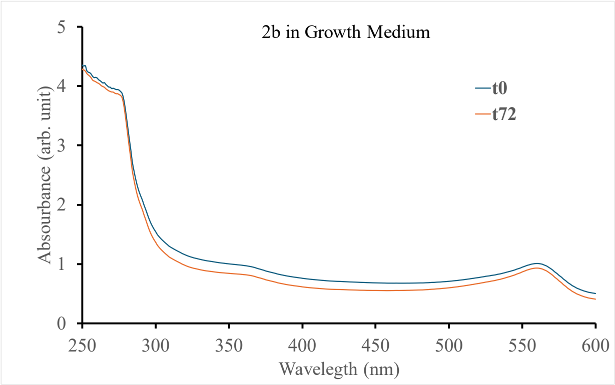

Figure S7G. UV-Vis Spectra of compound **2b** dissolved in cell media with 1% (v/v) DMSO immediately after sample preparation (blue trace, “T0”) and after 72 hours (yellow trace, “T72”); no observable changes were noticed between the two runs.

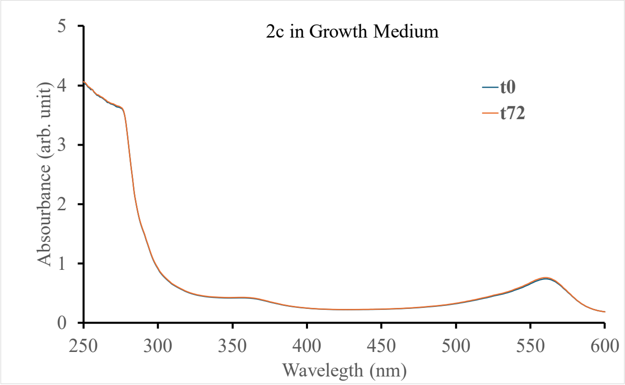

Figure S7H. UV-Vis Spectra of compound **2c** dissolved in cell media with 1% (v/v) DMSO immediately after sample preparation (blue trace, "T0") and after 72 hours (yellow trace, "T72"); no observable changes were noticed between the two runs.

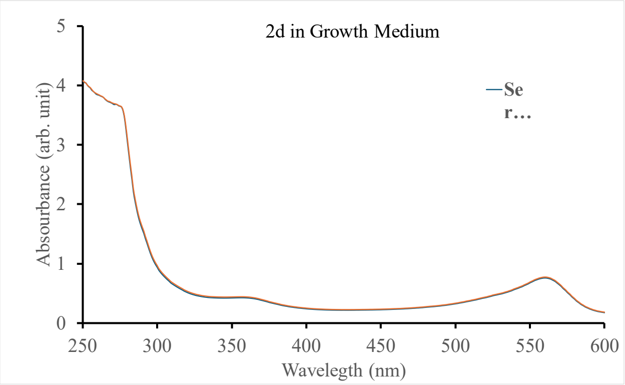

Figure S7I. UV-Vis Spectra of compound **2d** dissolved in cell media with 1% (v/v) DMSO immediately after sample preparation (blue trace, "T0") and after 72 hours (yellow trace, "T72"); no observable changes were noticed between the two runs.

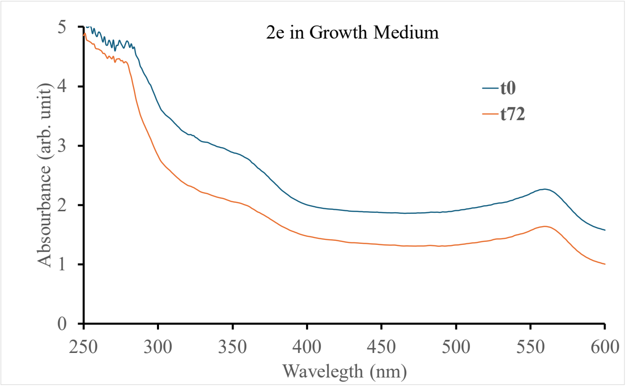

Figure S7J UV-Vis Spectra of compound **2e** dissolved in cell media with 1% (v/v) DMSO immediately after sample preparation (blue trace, “T0”) and after 72 hours (yellow trace, “T72”); no observable changes were noticed between the two runs.

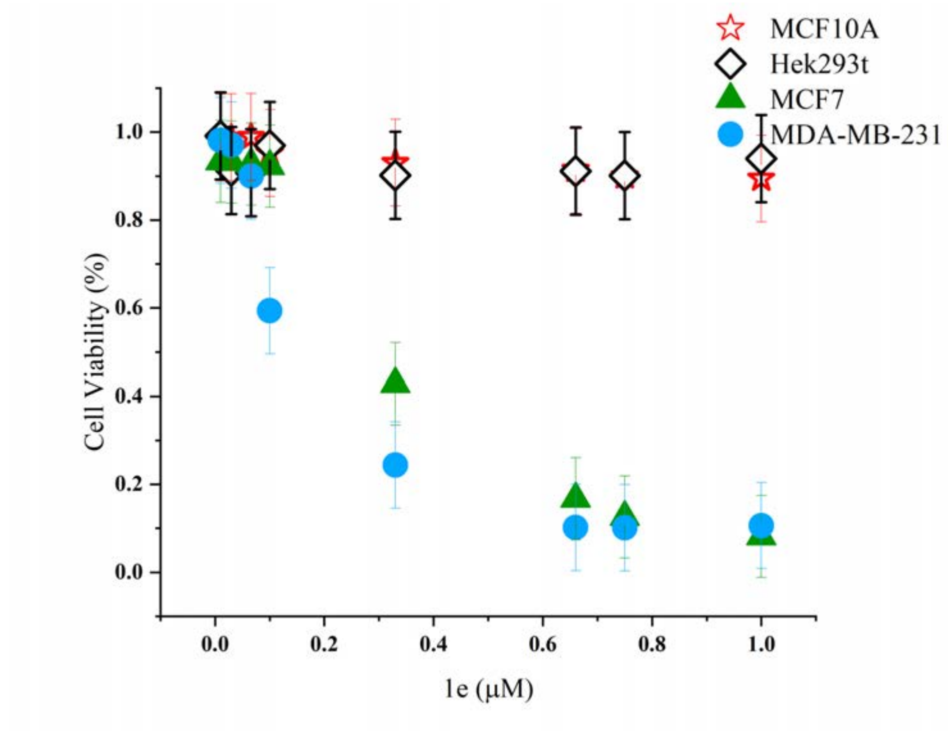

Figure S8A Eight-point dose-response curve for treatment with (TFP)Au(SMe$_2$pyrim) **1e** without DMSO.

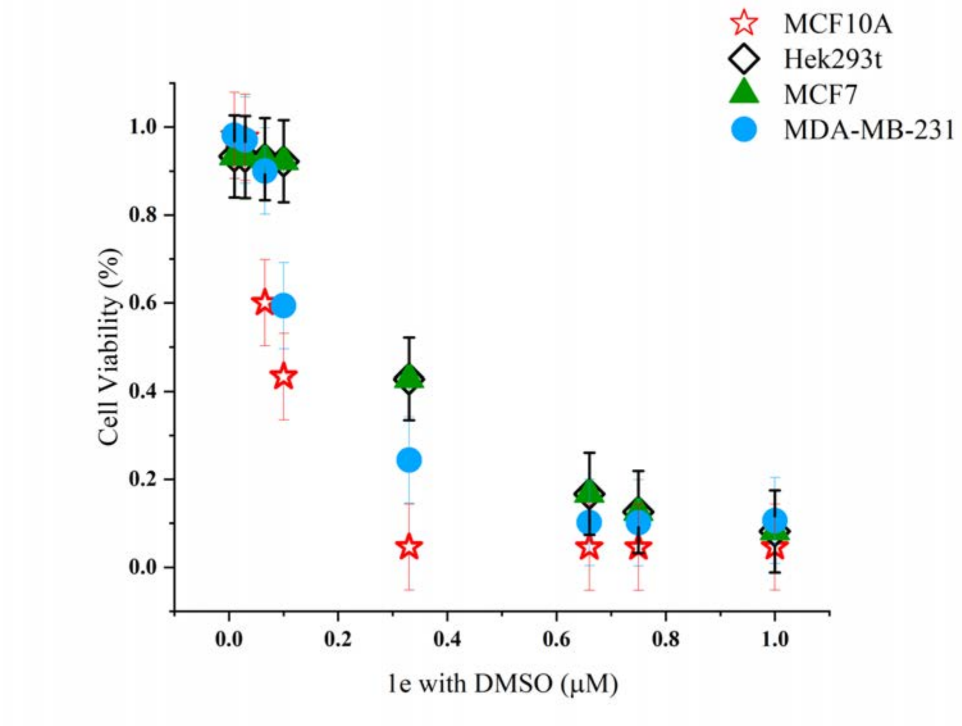

Figure S8B Eight-point dose-response curve for treatment with (TFP)Au(SMe_2pyrim) **1e** (in 1% v/v DMSO).

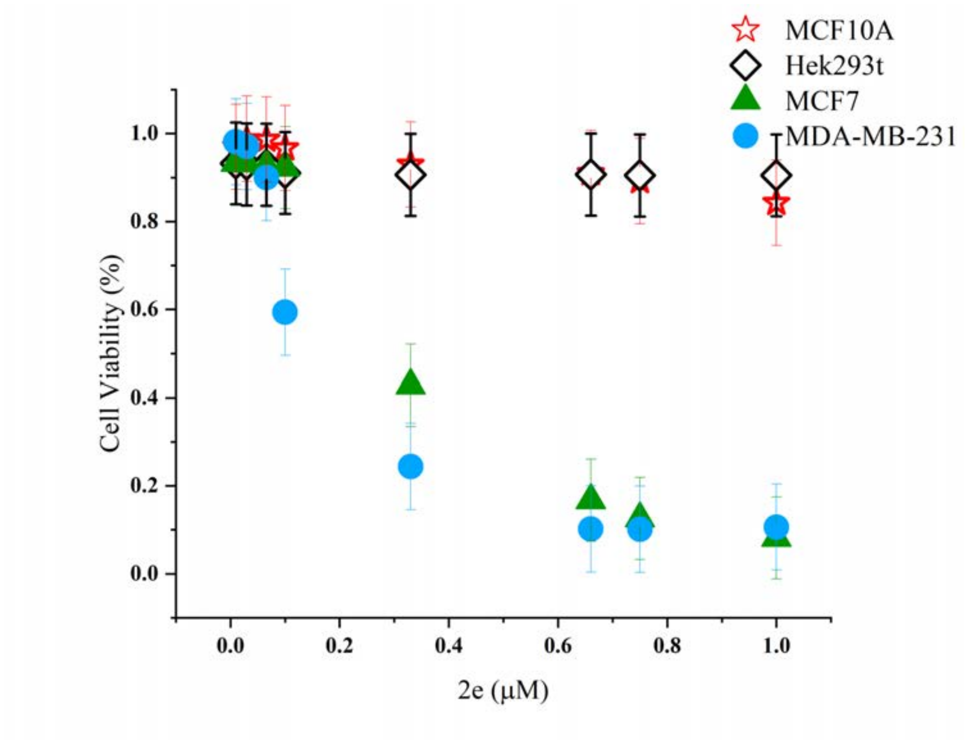

Figure S8C Eight-point dose-response curve for treatment with $(Ph_3P)Au(SMe_2pyrim)$ **2e** without DMSO.

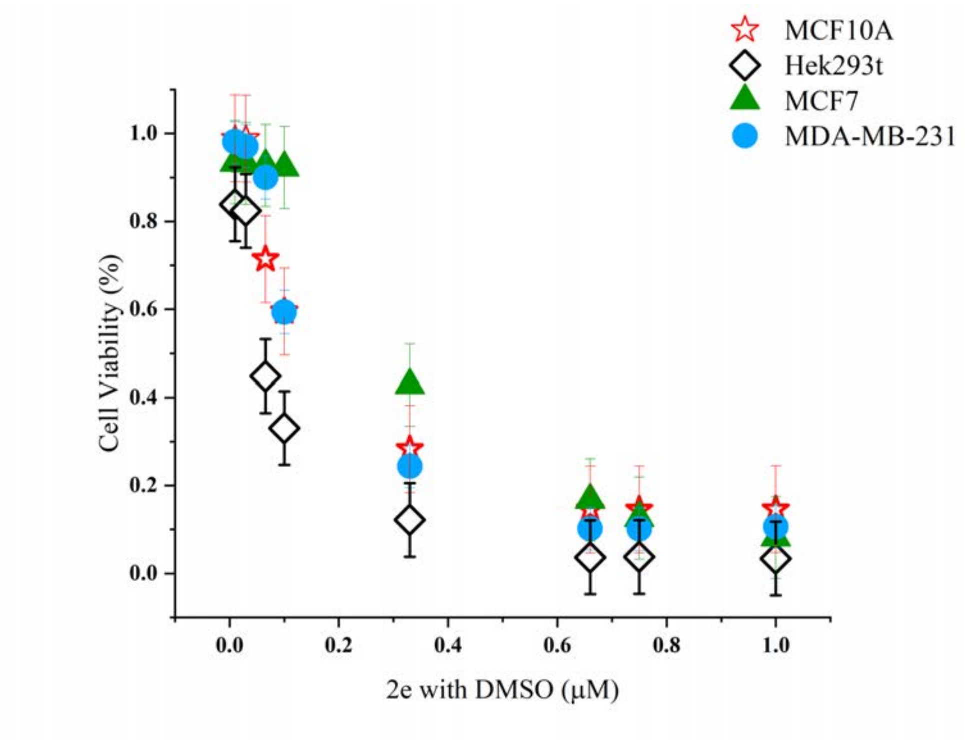

Figure S8D Eight-point dose-response curve for treatment with $(Ph_3P)Au(SMe_2pyrim)$ **2e** (in 1% v/v DMSO).

Table S1: Simplified molecular input line entry (SMILE) system structures for (**1a**-**1e** and **2a**-**2e**).

Compound Name	SMILES
**1a**	C1(S[Au][P](C2=CC=CO2)(C3=CC=CO3)C4=CC=CO4)=CC=CC=N1
**1b**	C1(S[Au][P](C2=CC=CO2)(C3=CC=CO3)C4=CC=CO4)=NC=CC=N1
**1c**	CC1=NC(S[Au][P](C2=CC=CO2)(C3=CC=CO3)C4=CC=CO4)=NC=C1
**1d**	FC(C1=NC(S[Au][P](C2=CC=CO2)(C3=CC=CO3)C4=CC=CO4)=NC=C1)(F)F
**1e**	CC1=NC(S[Au][P](C2=CC=CO2)(C3=CC=CO3)C4=CC=CO4)=NC(C)=C1
**2a**	C1(S[Au][P](C2=CC=CC=C2)(C3=CC=CC=C3)C4=CC=CC=C4)=CC=CC=N1
**2b**	C1(S[Au][P](C2=CC=CC=C2)(C3=CC=CC=C3)C4=CC=CC=C4)=NC=CC=N1
**2c**	CC1=NC(S[Au][P](C2=CC=CC=C2)(C3=CC=CC=C3)C4=CC=CC=C4)=NC=C1
**2d**	FC(C1=NC(S[Au][P](C2=CC=CC=C2)(C3=CC=CC=C3)C4=CC=CC=C4)=NC=C1)(F)F
**2e**	CC1=CC(C)=NC(S[Au][P](C2=CC=CC=C2)(C3=CC=CC=C3)C4=CC=CC=C4)=N1

Table S2. Elemental analyis data for **1a-1e** and **2a-2e**.

Cpd #	Formula	C (%) cal.	C (%) exp.	ΔC (%)	H (%) cal.	H (%) exp.	ΔH (%)	N (%) cal.	N (%) exp.	ΔN (%)
**1a**	$C_{17}H_{13}NO_3PSAu$. 0.5 H_2O	37.24	37.23	0.01	2.57	2.21	0.36	2.55	2.53	0.02
**1b**	$C_{16}H_{12}N_2O_3PSAu$	35.57	35.22	0.35	2.24	2.25	0.01	5.19	5.26	0.07
**1c**	$C_{17}H_{14}N_2O_3PSAu$	36.83	36.79	0.04	2.55	2.51	0.04	5.06	5.00	0.06
**1d**	$C_{17}H_{11}N_2F_3O_3PSAu$	33.56	33.15	0.41	1.82	1.67	0.15	4.61	4.43	0.18
**1e**	$C_{18}H_{16}N_2O_3PSAu$	38.04	37.40	0.64	2.84	2.70	0.14	4.93	4.78	0.15
**2a**	$C_{23}H_{19}NPSAu$. 0.25 CH_2Cl_2	47.28	47.21	0.07	3.33	3.18	0.15	2.37	2.42	0.01
**2b**	$C_{22}H_{18}N_2PSAu$. 0.5 H_2O	45.61	45.41	0.20	3.31	3.05	0.26	4.83	4.74	0.09
**2c**	$C_{23}H_{20}N_2PSAu$	47.28	47.06	0.22	3.45	3.21	0.24	4.80	4.89	0.09
**2d**	$C_{23}H_{17}N_2F_3PSAu$	43.27	42.97	0.30	2.68	2.35	0.33	4.39	4.06	0.33
**2e**	$C_{24}H_{22}N_2PSAu$	48.16	47.65	0.51	3.71	3.62	0.09	4.68	4.59	0.09

Experimental (exp.) values for elemental analyses of C, H, and N (columns 4, 7, and 10, respectively) were within 0.4-0.6% (indicated as percent differences ΔC, ΔH, and ΔN in columns 5, 8, and 11) of their calculated (cal.) values (columns 3, 6, and 9) for all compounds. Addition of partial solvent molecules (0.5 H_2O for **1a** and **2b**, and 0.25 CH_2Cl_2 for **2a**) is justified based on integration of the ^{1}H NMR spectra.

Table S3 (p.S64-65). Yields, melting points, and spectral assignments for **1a-1e**; **2a-2e**.

(TFP)Au(Spyridine) **1a***:* Yield = 200 mg (69 %). Melting points (M.P.) 134 °C d. ^{1}H NMR ($CDCl_3$) d 6.54 (m, 3H, H_{furyl}); 6.89 (m, 1H, H_{pyrid}); 7.28 (m, 3H, H_{furyl}); 7.34 (m, 1H, H_{pyrid}); 7.48 (m, 1H, H_{pyrid}); 7.78 (m, 3H, H_{furyl}); 8.28 (m, 1H, H_{pyrid}). ^{13}C NMR ($CDCl_3$) d 111.5, C_{furyl}; 118.5, C_{pyrid}; 125.2, C_{furyl}; 126.8, C_{pyrid}; 135.8, C_{pyrid}; 142.8, C_{furyl}; 148.4, C_{pyrid}; 149.9, C_{furyl}; 166.6, C_{pyrid}. ^{31}P{^{1}H}NMR ($CDCl_3$) d -24.2. FT-IR [ν, cm^{-1}] 691 (w), ν(S-C); 750 (vs), ν(P-C); 1006 (s), ν(Au-P); 1115 (w), 1325 (s), 1568 (vs), ν(C = N); 3105 (w), ν(C-H_{aryl}).

(TFP)Au(Spyrimidine) **1b**: Yield = 195 mg (67 %). M.P. 130 °C d. ^{1}H NMR ($CDCl_3$) d 6.53 (m, 3H, H_{furyl}); 6.74 (t, J_{HH} = 9.76 Hz, 1H, H_{pyrim}); 7.31 (m, 3H, H_{furyl}); 7.79 (m, 3H, H_{furyl}); 8.23 (d, J_{HH} = 4.68 Hz, 2H, H_{pyrim}). ^{13}C NMR ($CDCl_3$) d 111.6, C_{furyl}; 115.9, C_{pyrim}; 125.5, C_{furyl}; 141.8, C_{furyl}; 150.0, C_{furyl}; 156.8, C_{pyrim}; 180.4, C_{pyrim}. ^{31}P{^{1}H} NMR ($CDCl_3$) d -23.4. FT-IR [ν, cm^{-1}] 691 (w), ν(S-C); 751 (vs), ν(P-C); 1006 (s), ν(Au-P); 1117 (s), 1325 (s), 1570 (s), ν(C = N); 1406 (m), ν(C = C); 3105 (w), ν(C-H_{aryl}).

(TFP)Au(SMepyrimidine) **1c**: Yield = 219 mg (73 %). M.P. 138 °C d. ^{1}H NMR ($CDCl_3$) d 2.39 (s, 3H, H_{methyl}); 6.53 (m, 3H, H_{furyl}); 6.75 (d, 1H, H_{pyrim}); 7.32 (m, 3H, H_{furyl}); 7.79 (m, 3H, H_{furyl}); 8.23 (d, J_{HH} = 4.80 Hz, 1H, H_{pyrim}). ^{13}C NMR ($CDCl_3$) d 24.3, C_{methyl}; 111.5, C_{furyl}; 115.5, C_{pyrim}; 125.3, C_{furyl}; 142.0, C_{furyl}; 149.9, C_{furyl}; 156.3, C_{pyrim}; 166.7, C_{pyrim}; 179.8, C_{pyrim}; ^{31}P{^{1}H} NMR ($CDCl_3$) d -23.8. FTIR [ν, cm^{-1}] 542 (w), ν(Au-S); 692 (w), ν(S-C); 753 (vs), ν(P-C); 1009 (s), ν(Au-P); 1118 (s), 1325 (s), 1566 (s), ν(C = N); 3079 (w), ν(C-H_{aryl}).

(TFP)Au(SCF$_3$pyrimidine) **1d**: Yield = 225 mg (69 %). M.P. 145 °C d. ^{1}H NMR ($CDCl_3$) d 6.55 (m, 3H, H_{furyl}); 7.16 (d, J_{HH} = 5.20 Hz, ^{1}H, H_{pyrim}); 7.29 (m, 3H, H_{furyl}); 7.80 (m, 3H, H_{furyl}); 8.60 (d, J_{HH} = 5.20 Hz, 1H, H_{pyrim}). ^{13}C NMR ($CDCl_3$) d 110.9, C_{CF3}; 111.7, C_{furyl};; 125.7, C_{furyl}; 141.5, C_{furyl}; 142.4, C_{pyrim}; 150.0, C_{furyl}; 154.8, C_{pyrim}; 158.9, C_{pyrim}; 228.6, C_{pyrim}. ^{31}P{^{1}H} NMR ($CDCl_3$) d -23.4. FT-IR [ν, cm^{-1}] 553 (s), ν(Au-S); 665 (w), ν(S-C); 754 (vs), ν(P-C); 1005 (s), ν(Au-P); 1115 (s), 1325 (s), 1550 (s), ν(C = N); 3115 (w), ν(C-H_{aryl}).

(TFP)Au(SMe$_2$pyrimidine) (**1e**) Yield = 225 mg (73%). M.P. 145 °C d. ^{1}H NMR ($CDCl_3$) d 2.34 (s, 6H, H_{methyl}); 6.54 (m, 3H, H_{furyl}); 6.80 (s, 1H, H_{pyrim}); 7.31 (m, 3H, H_{furyl}); 7.79 (m, H_{furyl}). ^{13}C NMR ($CDCl_3$) d 24.1, C_{methyl}; 114.7, C_{furyl}; 129.2, C_{furyl}; 129.7, C_{pyrim}; 131, 7, C_{fuyl}; 134.4, C_{furyl}; 166.0, C_{pyrim}; 179.6, C_{pyrim}. ^{31}P{^{1}H} NMR ($CDCl_3$) d -24.1. FT-IR [ν, cm^{-1}] 543 (s), ν(Au-S); 693 (w), ν(S-C); 749 (vs), ν(P-C); 1006 (s), ν(Au-P); 1124 (s), 1574 (s), ν(C = N); 3096 (s), ν(C-H_{aryl}).

(Ph$_3$P)Au(Spyridine) **2a**: Yield = 200 mg (65%). M.P. 190 °C d. ^{1}H NMR ($CDCl_3$) d 6.85 (t, J_{HH} = 12.40 Hz, 1H, H_{pyrid}); 7.30 (t, J_{HH} = 17.20 Hz, 1H, H_{pyrid}); 7.48 (d, 1H, H_{pyrid}), 7.44-7.52 (m, 9H, H_{phenyl}); 7.58-7.64 (m, 6H, H_{phenyl}); 8.28 (d, J_{HH} = 4.28 Hz, 1H, H_{pyrid}). ^{13}C NMR $CDCl_3$) d 118.3, C_{pyrid}; 126.9, C_{pyrid}; 129.2, C_{phenyl}; 130.1, C_{phenyl}; 131.8, C_{phenyl}; 134.4, C_{phenyl}; 135.5, C_{pyrid}; 148.6, C_{pyrid}; 167.1, C_{pyrid}. ^{31}P{^{1}H} NMR ($CDCl_3$) d 38.2. FT-IR [ν, cm^{-1}] 689 (w), ν(S-C); 757 (vs), ν(P-C); 1008 (s), ν(Au-P); 1117 (s), 1328 (s) 1570 (s), ν(C = N); 3115 (s), ν(C-H_{aryl}

(Ph$_3$P)Au(Spyrimidine) **2b**: Yield = 210 mg (68%). M.P. 210 °C d. ^{1}H NMR ($CDCl_3$) d 6.84 (t, J_{HH} = 10.40 Hz, 1H, H_{pyrim}); 7.48-7.58 (m, 9H, H_{phenyl}); 7.62-7.68 (m, 9H, H_{phenyl}); 8.34 (d, J_{HH} =

5.20 Hz, 2H, H_{pyrim}). ^{13}C NMR ($CDCl_3$) d 115.6, C_{pyrim}; 129.2, C_{phenyl} ; 130.1, C_{phenyl}; 131.8, C_{phenyl}; 134.6, C_{phenyl}; 156.7, C_{pyrim}; 180.9, C_{pyrim}. $^{31}P\{^1H\}$ NMR ($CDCl_3$) d 38.1. FTIR [ν, cm^{-1}] 691(w), ν(S-C); 753 (vs), ν(P-C); 1006 (s), ν(Au-P); 1115 (s), 1328 (s), 1568 (s), ν(C = N); 3105 (w), ν(C-H_{aryl}).

(Ph_3P)Au(SMepyrimidine) **2c:** Yield = 195 mg (62 %). M.P. 208 °C d. ^1H NMR ($CDCl_3$) d 2.37 (s, 3H, H_{methyl}); 6.71 (d, J_{HH} = 4.80 Hz, 1H, H_{pyrim}); 7.46-7.64 (m, 15H, H_{phenyl}); 8.21 (d, J_{HH} = 4.80 Hz, 1H, H_{pyrim}). ^{13}C NMR ($CDCl_3$) d 24.7, C_{methyl}; 115.5, C_{pyriml}; 129.5, C_{phenyl}; 130.5, $C_{phenyl;}$; 132.1, C_{phenyl}; 134.9, C_{phenyl}; 156.6, C_{pyrim}; 166.7, C_{pyrim};180.5 C_{pyrim}. $^{31}P\{^1H\}$ NMR ($CDCl_3$) d 38.3. FTIR [ν, cm^{-1}] 691 (w) ν(S-C); 750 (vs), ν(P-C); 1009 (s), ν(Au-P); 1125 (s), 1326 (s), 1556 (s) ν(C = N); 2965 (w), ν(C-H_{aryl}); 2929 (w), ν(C-H_{alkyl}).

(Ph_3P)Au(SCF_3pyrimidine) **2d**: Yield = 230 mg (67 %). M.P. 205 °C d. ^1H NMR ($CDCl_3$) d 7.13 (d, J_{HH} = 4.80 Hz, 1H, H_{pyrim}); 7.45-7.56 (m, 9H, H_{phenyl}); 7.58-7.66 (m, 6H, H_{phenyl}); 8.57 (d, J_{HH} = 5.20 Hz, 1H, H_{pyrim}). ^{13}C NMR ($CDCl_3$) d 110.5, C_{CF3}; 119.0 C_{pyrim}; 121.7, C_{pyrim}; 129.0, C_{phenyl}; 131.7, C_{phenyl}; 134.1, C_{phenyl}; 154.3, C_{pyrim}; 158.5, $C_{phenyll}$; 182.3, C_{pyrim}. $^{31}P\{^1H\}$ NMR ($CDCl_3$) d 38.2. FTIR [ν, cm^{-1}] 691 (w), ν(S-C); 753 (s), ν(P-C); 1009 (s), ν(Au-P); 1118 (s), 1326 (s), 1554 (s), ν(C = N); 3108 (w), ν(C-H_{aryl}).

(Ph_3P)Au(SMe_2pyrimidine) **2e**: Yield = 316 mg (98%). M.P. 245 °C d. ^1H NMR ($CDCl_3$) d 2.32 (s, 6H, H_{methyl}); 6.59 (s, 1H, H_{pyrim}); 7.43-7.53 (m, 9H, H_{phenyl}); 7.60-7.67 (m, 6H, H_{phenyl}). ^{13}C NMR ($CDCl_3$) d 24.1, C_{methyl}; 114.7, C_{pyrim}; 129.2, C_{phenyl}; 129.8, C_{pyrim}; 130.3, C_{phenyl}; 131.7, C_{phenyl}; 134.4, C_{phenyl}; 166.0, C_{pyrim}. $^{31}P\{^1H\}$ NMR ($CDCl_3$) d 38.1. FTIR [ν, cm^{-1}] 690 (w), ν(S-C); 747 (s), ν(P-C); 1098 (s), ν(Au-P); 1335 (s), 1567 (s), ν(C = N); 2984(w), ν(C-H_{alkyl}); 3082 (w), ν(C-H_{aryl}).

Table S4. Crystal data for the structure determination of **1d**

Formula	$C_{17}H_{11}AuF_3N_2O_3PS$
Formula Weight	608.27
Crystal System	*Monoclinic*
Space group	*P2₁/c*
a (Å)	11.2043(5)
b (Å)	14.0447(6)
c (Å)	12.0827(5)
α (°)	90
β (°)	94.369(2)
γ (°)	90
V (Å^3)	1895.82(14)
Z	4
r(calc) (g/cm^3)	2.131
m (mm^{-1})	8.004
F(000)	1152.0
Crystal Size(mm)	0.66 × 0.31 × 0.26
T (K)	296.15
Radiation wavelength (Å)	0.71073
θ (Min-Max) (°)	7.296 - 57.4
*hkl*range	–15 to 15, –18 to 18 and –16 to 16
Reflections collected	32121
Independent reflections	4880
R_{int}	0.0989
Parameters	280
GOF on F^2	1.081
R_1[a] /wR_2[b] (I > 2σ(I))	0.0369/0.0655
R_1[a] /wR_2[b] (all)	0.00771/0.0782

[a] R_1= [Σ abs(abs(Fo) - abs(Fc))]/ [Σ abs(Fo)]

[b] $wR_2 = [\Sigma(w(Fo^2 - Fc^2)^2)/ \Sigma[w(Fo^2)^2]^{1/2}$

Table S5: Optimized LC gradient used for LC MS purity and stability assessments of **1a-1e** and **2a-2e**, examined at an LC flow rate of 0.5 μL/min; LC solvents A and B refer to water and acetonitrile, respectively.

Time (min.)	%A	%B
Initial	99	1
5	99	1
30	1	99
35	1	99
40	99	1
45	99	1
