# Supplementary figures and images for: Evaluating the Antiproliferative Effects of Tri(2-Furyl)- and Triphenylphosphine-Gold(I) Pyridyl- and Pyrimidine-Thiolate Complexes
Source: Biomolecules. 2026 Jan 15;16(1):154. doi: 10.3390/biom16010154 (PMC12839414; doi:10.3390/biom16010154)

**Series 1 - Tri(furyl)phosphine (TFP)**

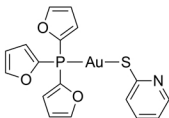

**1a**

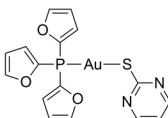

**1b**

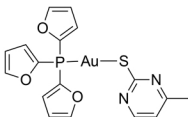

**1c**

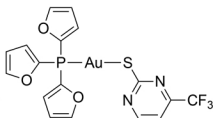

**1d**

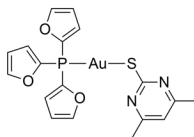

**1e**

**Series 2 - Triphenylphosphine (Ph<sub>3</sub>P)**

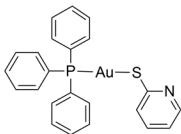

**2a**

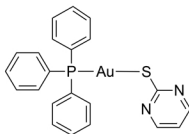

**2b**

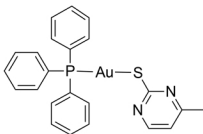

**2c**

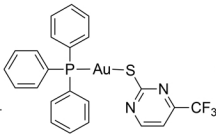

**2d**

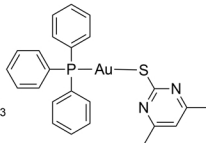

**2e**

Supplement: Supplementary file 1 [file biomolecules-16-00154-s001.zip › Scheme 1 (1200).pdf]
